# Particle-Associated Differ from Free-Living Bacteria in Surface Waters of the Baltic Sea

**DOI:** 10.3389/fmicb.2015.01297

**Published:** 2015-12-01

**Authors:** Angelika Rieck, Daniel P. R. Herlemann, Klaus Jürgens, Hans-Peter Grossart

**Affiliations:** ^1^Leibniz-Institute of Freshwater Ecology and Inland FisheriesStechlin, Germany; ^2^Leibniz Institute for Baltic Sea Research WarnemündeRostock, Germany; ^3^Institute of Biochemistry and Biology, University of PotsdamPotsdam, Germany

**Keywords:** microbial communities, microbial diversity, particle-associated and free-living bacteria, Baltic Sea, salinity gradient, seasons, 454-pyrosequencing

## Abstract

Many studies on bacterial community composition (BCC) do not distinguish between particle-associated (PA) and free-living (FL) bacteria or neglect the PA fraction by pre-filtration removing most particles. Although temporal and spatial gradients in environmental variables are known to shape BCC, it remains unclear how and to what extent PA and FL bacterial diversity responds to such environmental changes. To elucidate the BCC of both bacterial fractions related to different environmental settings, we studied surface samples of three Baltic Sea stations (marine, mesohaline, and oligohaline) in two different seasons (summer and fall/winter). Amplicon sequencing of the 16 S rRNA gene revealed significant differences in BCC of both bacterial fractions among stations and seasons, with a particularly high number of PA operational taxonomic units (OTUs at genus-level) at the marine station in both seasons. “Shannon and Simpson indices” showed a higher diversity of PA than FL bacteria at the marine station in both seasons and at the oligohaline station in fall/winter. In general, a high fraction of bacterial OTUs was found exclusively in the PA fraction (52% of total OTUs). These findings indicate that PA bacteria significantly contribute to overall bacterial richness and that they differ from FL bacteria. Therefore, to gain a deeper understanding on diversity and dynamics of aquatic bacteria, PA and FL bacteria should be generally studied independently.

## Introduction

Nutrient availability, pH, temperature, and salinity have been shown to be major environmental drivers for community structure and activities of aquatic organisms. Temporal and spatial gradients in environmental factors shape community composition and metabolic activities of aquatic organisms, and hence biogeochemistry of aquatic ecosystems. Although macroscopic organic aggregates (>100 μm) play a crucial role in the ocean's carbon cycle (Fowler and Knauer, 1986; Alldredge and Silver, 1988; Simon et al., 2002), and particle-associated (PA) bacteria colonize nearly all types of particulate organic matter (POM), studies in microbial ecology traditionally focus on total or exclusively free-living (FL) bacteria (Grossart et al., 2010). This is in contrast to findings indicating that macroscopic organic aggregates produced by aggregation of various organic material, i.e., marine snow (Simon et al., 2002) or lake snow (Grossart and Simon, 1993) are densely colonized by bacteria. They represent nutrient rich “hotspots” of microbial activity even in oligotrophic environments (e.g., Simon et al., 2002; Grossart, 2010). Differences in hydrodynamic conditions and primary production, however, may lead to changes in organic matter quantity and quality resulting in different aggregation dynamics and consequently of microbial particle colonization. Ortega-Retuerta et al. (2013) suggested that bacterial community composition (BCC) of the PA fraction in the ocean is rather determined by particle quality than by their quantity, since differences between PA and FL bacteria are not always correlated to POM concentrations, whereas the ratio between PA and FL bacteria relates well to suspended particulate matter (SPM) quality (Doxaran et al., 2012). Such differences in BCC and dynamics of both bacterial fractions indicate that PA and FL bacteria should be regarded as independent components of a bacterial assemblage to better understand the bacterial response to a changing environment. Yet, many studies in aquatic microbial ecology do not distinguish between PA and FL bacteria or remove most PA bacteria with particles from the sample when using pre-filtration steps.

The Baltic Sea is an ideal model system to address general differences in BCC and dynamics of both bacterial fractions at different salinities. It is one of the largest brackish (mesohaline) ecosystems in the world where salinity declines along a ca. 2000 km long horizontal gradient from salinities of around 32 in the Kattegat to 2 in the northern Bothnian Bay. The narrow and shallow Danish straits limits water exchange with the North Sea resulting in a water residence time of the surface waters of ca. 25–30 years (Kautsky and Kautsky, 2000) and relatively stable horizontal and vertical salinity gradients. Salinity has been shown to shape the distribution pattern of macroorganisms, with higher organisms being either adapted to freshwater or marine systems resulting in a relatively lower diversity in the freshwater-marine transition zone (Remane, 1934; Telesh and Khlebovich, 2010). Protozoa, however, did not show a clear salinity-dependent distribution pattern (Telesh and Khlebovich, 2010) and bacterial communities in the Baltic Sea revealed only little differences in α-diversity along the salinity gradient (Herlemann et al., 2011). In contrast, BCC greatly varies along the horizontal and vertical salinity gradients in the Baltic Sea (Edwards and John Pojeta, 1997; Holmfeldt et al., 2009; Herlemann et al., 2011). None of these studies, however, distinguished between PA and FL bacteria communities, and at present we do not know whether the diversity of PA bacteria is shaped by the salinity gradient and to what extent they differ from FL communities.

The majority of studies differentiating between PA and FL bacteria has been either conducted in marine or in freshwater habitats (Simon et al., 2002; Ghiglione et al., 2007, 2009; Fontanez et al., 2015). Only a few studies compared BCC of both bacterial fractions at different salinities in marine, brackish and freshwater ecosystems (e.g., Crump et al., 1999, 2004; Ortega-Retuerta et al., 2013; Bižić-Ionescu et al., 2014; Simon et al., 2014). Nevertheless, all of these studies found general differences in BCC between habitats and bacteria fractions. Whereas some studies indicated a high specialization of bacteria on particles (Crump et al., 1999; Rösel and Grossart, 2012) others point to a high exchange between FL and PA bacteria fractions (Hollibaugh et al., 2000; Ghiglione et al., 2007). Since enzymatic capabilities greatly differ among various bacterial phyla and life-styles (Martinez et al., 1996; Lyons and Dobbs, 2012), knowledge about BCC of both PA and FL bacteria fractions provides hints on metabolic differences. For example, Ganesh et al. (2015) focusing on dissimilatory processes of the nitrogen cycle in the oxygen minimum zone of the Eastern Tropical North Pacific revealed 8- to 15-fold higher bacterial gene counts in the FL fraction, which was mainly related to anammox-associated transcripts.

In particular, little is known about PA bacteria from the marine-freshwater transition zone. Interestingly, Crump et al. (1998) found different PA bacteria at the transition from the river to the estuary and coastal sites, whereas FL bacteria remained similar. Garneau et al. (2009) found the strongest differences in bacterial fractions at the estuarine station with the highest POM content. Moreover, Ortega-Retuerta et al. (2013) have analyzed various samples from a river, the coastal and open ocean and found significant differences in BCC of total bacteria between samples at different salinities. However, BCC of PA and FL bacteria differed only in the open ocean, although particle concentration was much higher in the riverine and coastal samples. The authors also found a generally higher diversity for PA bacteria (Shannon, Simpson, and Chao indices) than FL. We hypothesized that (1) PA bacteria significantly contribute to overall bacterial richness, and (2) that salinity has a strong impact on the composition of PA bacteria. During two seasons, we assessed PA and FL BCC at three stations in the Baltic Sea with different salinities using 454-pyrosequencing of partial 16S rRNA genes (spanning variable regions V3 and V4). In summer, we simultaneously measured the cell-specific bacterial protein production (csBPP) by ^14^C-leucine incorporation. Our results indicate that PA bacteria substantially contribute to overall bacterial α- and β-diversity at all stations in both sampling seasons (summer and fall/winter). BCC substantially differed among both bacterial fractions, whereby α- and β-diversity of PA and FL bacteria varied with season and salinity. The highest bacterial α-diversity occurred in the PA fraction of the marine station in fall/winter. Although PA bacteria contributed rather little to total bacterial abundance, csBPP rates of PA were significantly higher than FL bacteria. This suggests that PA bacteria represent an important component of overall bacterial α- and β-diversity and presumably activity in the Baltic Sea.

## Materials and methods

### Site description and field work

**Figure 1:**
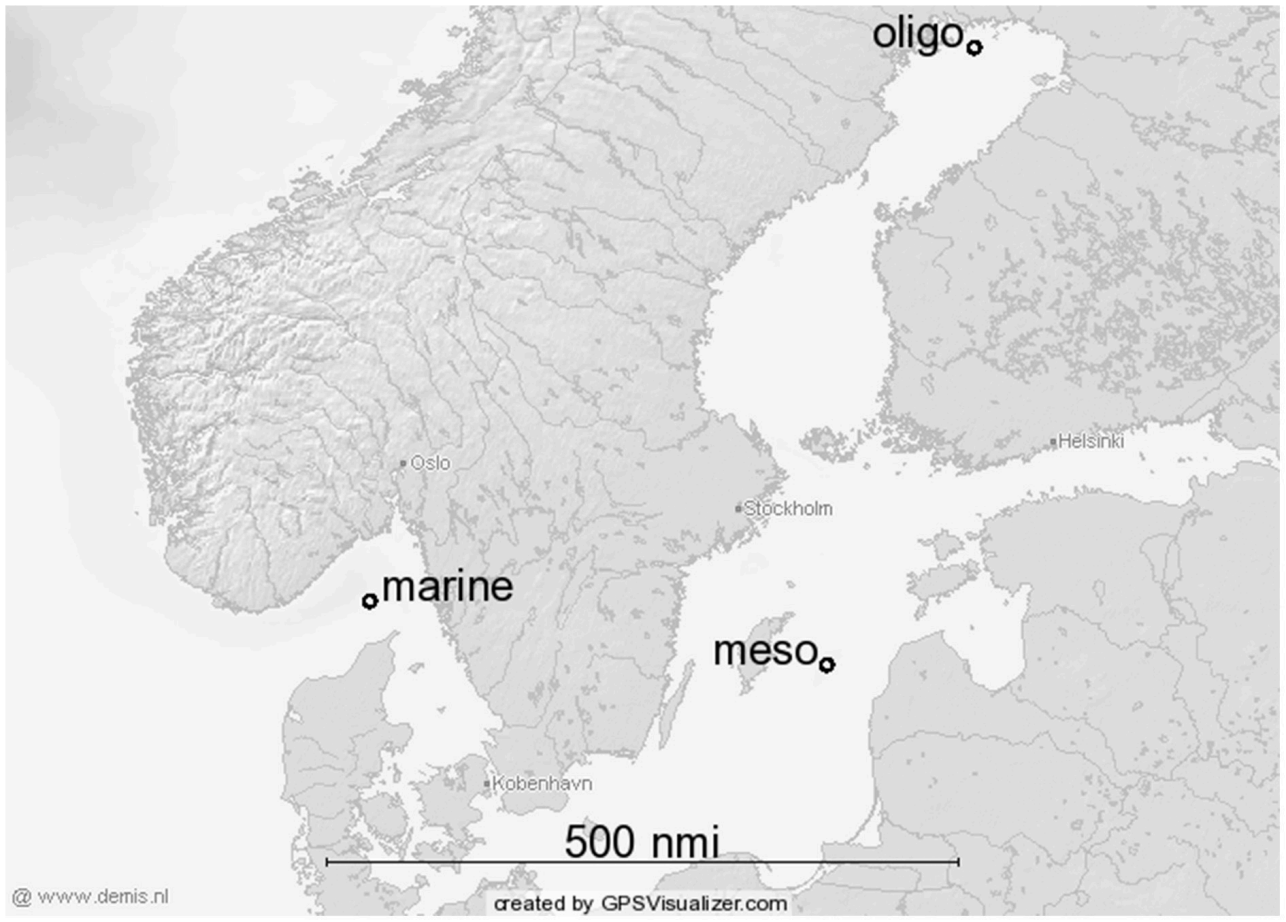
**Study area (Baltic Sea) and station locations**. Dots represent sampling stations considered as “marine” (mar), “mesohaline” (meso) and “oligohaline” (oligo) station.

The sampling was performed in the scope of the ATKiM (“Abbaubarkeit von arktischem, terrigenem Kohlenstoff im Meer”) project during two cruises (M86-1, M87-3) of the RV Meteor in the Baltic Sea: (i) November/December 2011 (“fall/winter”) and (ii) May/June 2012 (“summer”). Sampling locations (Figure 1) were characterized by differences in salinity and defined as marine (Skagerrak, salinity ≈ 30-34; N 58°07′59.88′′, E010°0′0′′), mesohaline (Gotland Basin, salinity ~ 7; N57°18′20.52′′; E020°04′41.5′′) and oligohaline (Bothnian Bay, salinity ≈2.7; N65°26′42′′, E023°17′53.88′′). Surface water of the three different sampling stations was collected with a 400 dm^3^ stainless steel water sampler (Hydrobios, Kiel, Germany) from which triplicate samples were taken (representing “technical replicates”). The samples were pre-filtrated through a 100 μm mesh filter to exclude large zooplankton and then directly transferred into 25 L polyethylene canisters from which subsamples were taken at 10°C for further analyses. The bacteria collected on the 5-μm filter were considered to represent the PA fraction and the bacteria collected on the 0.2−μm filters the FL fraction.

### Chemical parameters

Dissolved inorganic phosphate, nitrate, nitrite, silicate, and ammonium were analyzed following standard methods (Rohde and Nehring, 1979; Grasshoff et al., 1983). All samples were filtered through pre-combusted GF/F filters (pore size 0.65 μm; Whatman, Dassel, Germany) and hydrolyzed at 220°C, except for ammonia analysis. Phosphate, nitrate, nitrite, and silicate were analyzed by using a continuous-flow analyzer “FLOWSYS” (Alliance Instruments GmbH, Ainring, Germany). Ammonium concentration was determined by a UV mini 1240 photometer (Shimadzu).

For particulate organic carbon (POC), five-hundred ml-samples were filtered through pre-combusted (500°C, 3 h) GF/F filters (Whatman, Dassel, Germany). Filters were dried for 4 h at 50°C in prewashed petri dishes and stored in a desiccator until analysis. The samples were analyzed by using an organic elemental analyzer “Flash EA 1112” coupled to an isotope ratio mass spectrometer “Finnigan MAT V” (Thermo Fisher Scientific Inc., Waltham, USA) in the stable isotope laboratory of the “Museum für Naturkunde”, Berlin, Germany.

Dissolved organic carbon (DOC) content was determined by filtering 30 ml per sample through pre-combusted (500°C, 3 h) GF/F filters (Whatman, Dassel, Germany) into high-density polyethylene [HDPE] vials which were kept frozen until analysis. For analysis, the “fall/winter” samples were acidified with 2 M HCl until the pH was below 4 in order to remove all inorganic carbon. DOC samples were then measured in duplicates on a Multi N/C 3100 Analyzer (Jena Analytics, Germany) by applying non-dispersive infrared detection (NDIR) after combustion. The “summer” samples were acidified to pH 2 with hydrochloric acid and measured in duplicates per sample and in triplicates per station by an organic carbon analyzer “TOC-VCPH and TNM-1” (Shimadzu Deutschland GmbH, Duisburg, Germany). For both analyses, an internal standard of potassium hydrogen phthalate (5 mg L^−1^) was used.

### Bacterial enumeration

For enumeration, bacterial samples were fixed with formaldehyde (2% v/v) and stored at 4°C. The following filtration was done within 4 weeks (Buesing, 2005). All samples (30 ml) were sequentially filtered through a 5.0 μm polycarbonate membrane (Ø 25 mm; Whatman, Dassel, Germany) for PA bacteria, and 5 ml of the filtrate were sieved through a 0.2 μm filter for FL bacteria. All filters were air-dried and stored at −20°C until microscopy analysis (Grossart et al., 2005). For counting FL bacteria, the bacteria were stained for 5 min with 1 μg ml^−1^ DAPI (4′,6-diamidino-2-phenylindole) (AppliChem GmbH, Darmstadt, Germany) and the PA bacteria with 1:1000 SybrGold (LifeTechnologies, Carlsbad, USA). Both dyes were diluted in Citifluor AF1 mounting medium (Citifluor Ltd, London, UK) which was added directly on the filter before covering with the cover slide. All samples were analyzed with an epifluorescence microscope “Axio Imager.Z1” (Zeiss, Jena, Germany) at 630 x magnification with an automated cell counting approach by preparing pictures of 25–40 stacks at intervals of 0.3 μm (Zeder et al., 2011). The image analysis was performed with ImageJ (Abràmoff et al., 2004) after Massana et al. (1997). Percentages of particle area were analyzed at 200x and 630x magnification which were used to relate PA bacteria counts to sample volume.

### Bacterial protein production (BPP) and respiration

BPP was measured for the summer samples by incorporation of L-^14^C-leucine (Hartmann Analytic, Braunschweig, Germany) according to Simon and Azam (1989). For each station, six samples and two negative controls (2% v/v formaldehyde) were inoculated with a final concentration of 50 μCi to ensure the uptake of L-^14^C-leucine of FL as well as PA bacteria. The incubation was performed in the dark at 10°C and stopped after 1 h by adding formaldehyde (2% v/v). After fixation, samples were filtered sequentially onto 5.0 and 0.2 μm cellulose nitrate filter (Whatman, Dassel, Germany) for separating PA and FL bacteria, respectively. Proteins were extracted for 5 min in 5% trichloroacetic acid (TCA). Thereafter, the filters were washed twice with ultrapure water and then with ethanol (50%), before they were transferred into 20 ml scintillation vials and dissolved with 500 μl of ethyl actetate (15 min). Before counting, 10 ml of the scintillation cocktail “Ultima Gold” (Perkin-Elmer, Downers Grove, USA) was added and incubated overnight. The counting was determined as disintegrations per minute (DPM) on a liquid scintillation analyzer “TriCarb 2810 TR” (PerkinElmer, Downers Grove, USA). The produced protein amount was converted by a factor of 0.86 into carbon (Simon and Azam, 1989). Cell-specific BPP (csBPP) was calculated for both bacterial fractions based on their cell numbers.

Total bacterial respiration (BR) of the samples was measured by oxygen consumption of subsamples with a Sensor Dish Reader SDR 2 (PreSens, Regensburg, Germany) in air-tight glass vials using an optode system (Köster et al., 2012). For each sample, oxygen consumption rates were determined over 24 h twice per sample in the dark at 10°C. After a temperature correction by calculating the oxygen solubility at a given temperature using the Bunsen absorption coefficient α (T), the amount of consumed oxygen was converted into carbon by using 0.88 as a conversion factor (Fogg and Gerrard, 1990; Robinson et al., 1999). Bacterial growth efficiency (BGE) was calculated as BGE = BPP/ (BPP+BR).

### Bacterial community composition

For DNA extraction, a total of 36 samples were analyzed. Each sample of 0.5 L was sequentially filtered over a 5.0 μm Nucleopore TrackEtch polycarbonate membrane (Whatman, Dassel, Germany) for the PA-fraction and the total 5.0 μm-filtrate was sieved through a 0.22 μm Durapore membrane (Merck KGaA, Darmstadt, Germany) for the FL-fraction. Filters were shock-frozen in liquid nitrogen and stored at −80°C until use. DNA extraction was performed after Nercessian et al. (2005), using a Phenol-Chloroform protocol with cetyltrimethyl ammonium bromide (CTAB) as a complexing agent for polymeric substances.

All 36 samples were PCR amplified with 30 cycles using a 20 ng aliquot of each DNA sample for a 25 μl PCR reaction, the primer pair Bakt_341F (CCTACGGGNGGCWGCAG) and Bakt_805R (ACHVGGGTATCTAATCC) and the Fusion DNA Polymerase Herculase II (Agilent Technologies, Santa Clara, USA) according to Herlemann et al. (2011). Primers were tailed with sample-specific 5 bp barcodes a 454-adptor region, spanning variable regions V3 and V4 of the 16 S rRNA gene. PCR products were purified with Agencourt AMPure XP magnetic beads (Beckman Coulter GmbH, Krefeld, Germany), quantified with a Picogreen assay (LifeTechnologies, Carlsbad, USA), diluted and equally pooled. The bidirectional sequencing was performed on a 454 platform with Titanium Flex chemistry (Roche etc.). Our samples represent subsamples, from a run with 61 samples in total, which were sequenced at the Berlin Center for Genomics in Biodiversity Research (BeGenDiv, Berlin) with an average of 115,000 reads per run and accordingly on average 1885 reads per sample. After a brief quality check with Mothur (Schloss et al., 2009) (maximum number of N: 0, minimum sequence length: 150, and minimum exponential Q-score: 20) SILVAngs data analysis service (Yilmaz et al., 2013) was used to align the resulting sequences with the SILVA Incremental Aligner (SINA) and to remove contaminations of the dataset with non-rDNA sequences. SILVAngs performs an additional quality check by a minimal length cut-off (50 bases) as well as ambiguity and homopolymer check (max. 2%). After the quality control, identical reads were identified (dereplication), unique reads were clustered (OTUs), on a per sample basis, and the reference read of each OTU was classified. Dereplication and clustering was done using cd-hit-est version 3.1.2 (Li and Godzik, 2006). The clustering was performed with a minimum of 97% sequence identity to each other (pairwise distance and single linkage clustering). For each OTU clustering, the longest read was then used as a reference of this cluster for taxonomic classification. BLAST (version 2.2.28+) in combination with the SILVA SSURef dataset (release 119) was used to classify the sequences. The resulting classification of the reference sequence of a cluster was mapped to all members of the respective cluster as well as their replicates. Sequences having an average BLAST alignment coverage and alignment identity of less than 93% would be considered as unclassified. This method was first used by Klindworth et al. (2013) and Ionescu et al. (2012). PAST was used to generate rarefaction curves with an algorithm from Krebs (1989). To normalize the number of sequence reads between samples, a random subsampling according to the smallest n (430 sequences) was performed in R with the vegan package (Oksanen et al., 2011) and the rrarefy function. For taxonomic analysis the resulting reads were normalized based on OTUs representing the genus-level diversity (97% similarity). All analyses of α- and β-diversity were performed with the rarefied dataset.

### Sequences

All sequences are deposited in the European Nucleotide Archive under the study accession number: PRJEB9483 and the sample accession numbers: ERS742024 to ERS742056.

### Statistical analysis

All statistics was performed using the software R (R Core Team, 2013) and PAST (Hammer et al., 2012).

For α-diversity calculation of species richness CHAO1 index, Buzas and Gibson's Evenness [e^∧^H/S], Simpson Evenness [1-D] as well as Shannon [H] diversity indices were calculated with PAST. For β-diversity (BCC) Bray-Curtis dissimilarity matrixes were generated, visualized by non-metric multidimensional scaling (NMDS), unweighted-pair group method with arithmetic averages (UPMGA), and statistically verified by analysis of similarity (ANOSIM) with the R package vegan and the functions metaMDS, hclust (average) and anosim. To assess which taxa are primarily responsible for the observed differences between the sample groups a similarity percentage algorithm (SIMPER) was used with the function simper. For estimation of significant differences between PA and FL bacterial activities and richness a non-parametric Wilcoxon rank sum test for independent samples was performed in R.

## Results

### Site characteristics

All three sampling stations (Table 1) were characterized by similar water temperatures between the “summer” and “fall/winter” season, mainly because of exceptionally high temperatures in late autumn 2011. The oligohaline station is an exception, with an “unproductive” phase at our summer sampling, due to its late ice-coverage (until the end of spring).

**Table 1:** **Physical, chemical and biological parameters at different stations and seasons in the Baltic Sea**.

	**Summer**			**Fall/Winter**		
	**Marine**	**Mesohaline**	**Oligohaline**	**Marine**	**Mesohaline**	**Oligohaline**
Salinity	30.40	7.15	2.65	34.62	6.97	2.84
T [°C]	12.29	9.07	4.33	12.15	10.54	6.26
O_2_ [μM]	6.64	8.51	9.42	5.93	7.24	8.18
Chl*a* [μg L^−1^]	0.13	1.63	1.76	0.30	0.47	0.44
DOC [mg L^−1^]	1.97 (± 0.12)	4.48 (± 0.57)	5.55 (± 0.30)	1.20 (± 0.11)	3.63 (± 0.10)	5.20 (± 0.12)
POC [mg L^−1^]	0.09	0.39	0.13	0.08	0.13	0.19
NO_2_ [μM]	0.17	0.20	0.70	0.06	0.26	0.25
NO_3_ [μM]	0.00	0.00	5.04	0.36	0.14	4.09
PO_4_ [μM]	−0.02	0.09	0.00	0.29	0.16	0.02
SiO_4_ [μM]	0.05	9.64	43.14	3.70	4.95	39.00
NH_4_ [μM]	0.44	0.11	0.22	0.48	0.81	0.64
cells_PA [10^5^ cells ml^−1^]	0.26 (±0.06)	0.57 (± 0.13)	0.76 (± 0.21)	0.12 (± 0.069)	0.13 (± 0.08)	0.74 (± 0.03)
cells_FL [10^5^ cells ml^−1^]	5.16 (± 0.20)	7.37 (± 1.06)	16.55 (± 0.27)	5.19 (± 0.17)	11.30 (± 0.28)	11.70 (± 0.08)
cells_Tot [10^5^ cells ml^−1^]	5.42 (± 0.24)	7.94 (± 1.18)	17.31 (± 0.29)	5.31 (± 0.23)	11.40 (± 0.35)	12.50 (± 0.11)
PA_bac [%]	4.85	7.17	4.38	3.28	0.93	5.84
BPP_PA [μgC L^−1^h^−1^]	0.18 (± 0.02)	0.33 (± 0.07)	0.17 (± 0.07)	NA	NA	NA
BPP_FL [μgC L^−1^h^−1^]	0.12 (± 0.09)	0.32 (± 0.06)	0.25 (± 0.09)	NA	NA	NA
BPP_Tot [μgC L^−1^h^−1^]	0.30 (± 0.07)	0.65 (± 0.05)	0.34 (± 0.15)	NA	NA	NA
csBPP_PA [fgC cell^−1^h^−1^]	6.28	5.75	1.97	NA	NA	NA
csBPP_FL [fgC cell^−1^h^−1^]	0.23	0.43	0.15	NA	NA	NA
csBPP_Tot [fgC cell^−1^h^−1^]	0.55	0.81	0.24	NA	NA	NA
Respiration [μg C L^−1^ h^−1^]	1.97	1.49	1.09	NA	NA	NA
BGE [μB h^−1^]	0.11	0.30	0.28	NA	NA	NA

*Sample name abbreviations: T, temperature; O_2_, oxygen content; Chla, Chlorophyll a; DOC, dissolved organic carbon; POC, particulate organic carbon; NO_2_, nitrite concentration; NO_3_, nitrate concentration; PO_4_, phosphate concentration; SiO_4_, silicate concentration; NH_4_, ammonium concentration; PA, particle-associated; FL, free-living; Tot, total; bac, bacteria; BPP, bacterial protein production; csBP, cell specific bacterial production; BGE, bacterial growth efficiency*.

Beside its high salinity (>30), the marine station showed the highest temperature (~12°C) as well as the lowest DOC (~1–2 mg L^−1^), POC (~0.08 mg L^−1^), oxygen (~6–7 mg L^−1^) and SiO_4_ concentrations (3.7 μM and 0.05 μM in fall/winter and summer, respectively) (Table 1). Nitrate content was higher in fall/winter than in summer (0.36 μM and below detection limit, respectively). The same pattern occurred for dissolved phosphate (0.29 μM vs. below the detection limit). Consequently, Chl*a* concentration was higher in fall/winter than in summer (0.3 vs. 0.13 μg L^−1^), whereby fall/winter values were in the same range as for the two other sampling stations.

The mesohaline station showed large differences in POC and Chl*a* concentrations between both seasons and was characterized by a salinity of around 7 (Table 1). DOC and SiO_4_ concentrations were close to the average of what can be expected in the Baltic Sea (DOC: 3.6 and 4.48 mg L^−1^ as well as 4.95 and 9.64 mg L^−1^ SiO_4_ in fall/winter and summer, respectively). Oxygen varied in both seasons between 7.24 and 8.51 mg L^−1^. In summer, POC and Chl*a* values (0.39 mg L^−1^ and 1.63 μg L^−1^) were clearly higher than in the fall/winter season (0.13 mg L^−1^ and 0.47 μg L^−1^). In contrast, NO_3_, NH_4_, and PO_4_, which were either close to or below the detection limit in summer (0.00, 0.11, and 0.09 μM), were much higher in fall/winter (0.14, 0.81, and 0.16 μM).

The oligohaline station was characterized by the lowest salinity (~2.7) and temperature (4.33 and 6.26°C in fall/winter and summer, respectively), but by the highest DOC (>5 mg L^−1^), silicate (39 and 43 μM in fall/winter and summer, respectively), and oxygen concentrations (8.18 and 9.42 mgL^−1^ in fall/winter and summer, respectively) (Table 1). POC concentrations did not differ much between summer (0.13 mg L^−1^) and fall/winter (0.19 mg L^−1^). In summer, POC at the oligohaline station was lower than at the mesohaline station. Chl*a* concentration, however, increased from 0.44 μg L^−1^ in fall/winter to 1.76 μg L^−1^ in summer - similar to the mesohaline station. Dissolved nitrogen concentration was highest at the oligohaline station (4.0 μM in fall/winter and 5.0 μM in summer).

### Bacterial numbers and activity

At both sampling time points, the total number of bacterial cells (Table 1) was lowest at the marine station, and no trend in abundance was obvious between both seasons. Cell number was lower in summer at the mesohaline station, particularly for FL cells. The opposite trend occurred at the oligohaline station. Numbers of PA bacteria at the marine and mesohaline stations were higher in summer whereas those of the oligohaline station remained at the same high level in both seasons. Marine and oligohaline stations showed an average proportion of PA bacteria between 3 and 6% independent of season. The mesohaline station, however, showed a remarkably higher proportion of PA bacteria in summer (~1% in fall/winter vs. 7% in summer) in accordance with the higher POC concentration. In contrast to the relatively low abundance of PA bacteria, bacterial protein production (BPP) (only measured in summer) was almost in the same range as for FL bacteria. Cell specific bacterial protein production (csBPP) of all PA bacteria was significantly higher than of FL bacteria (~17 times) (Wilcoxon rank sum test: *W* = 36, *p* = 0.005). For all bacteria fractions, highest csBPP occurred at the marine station, followed by the mesohaline and oligohaline stations. In parallel to the rather low total BPP, community respiration was also lowest at the oligohaline station and calculated bacterial growth efficiencies of total bacteria varied between 11 and 30% (Table 1).

### Bacterial diversity

**Figure 2:**
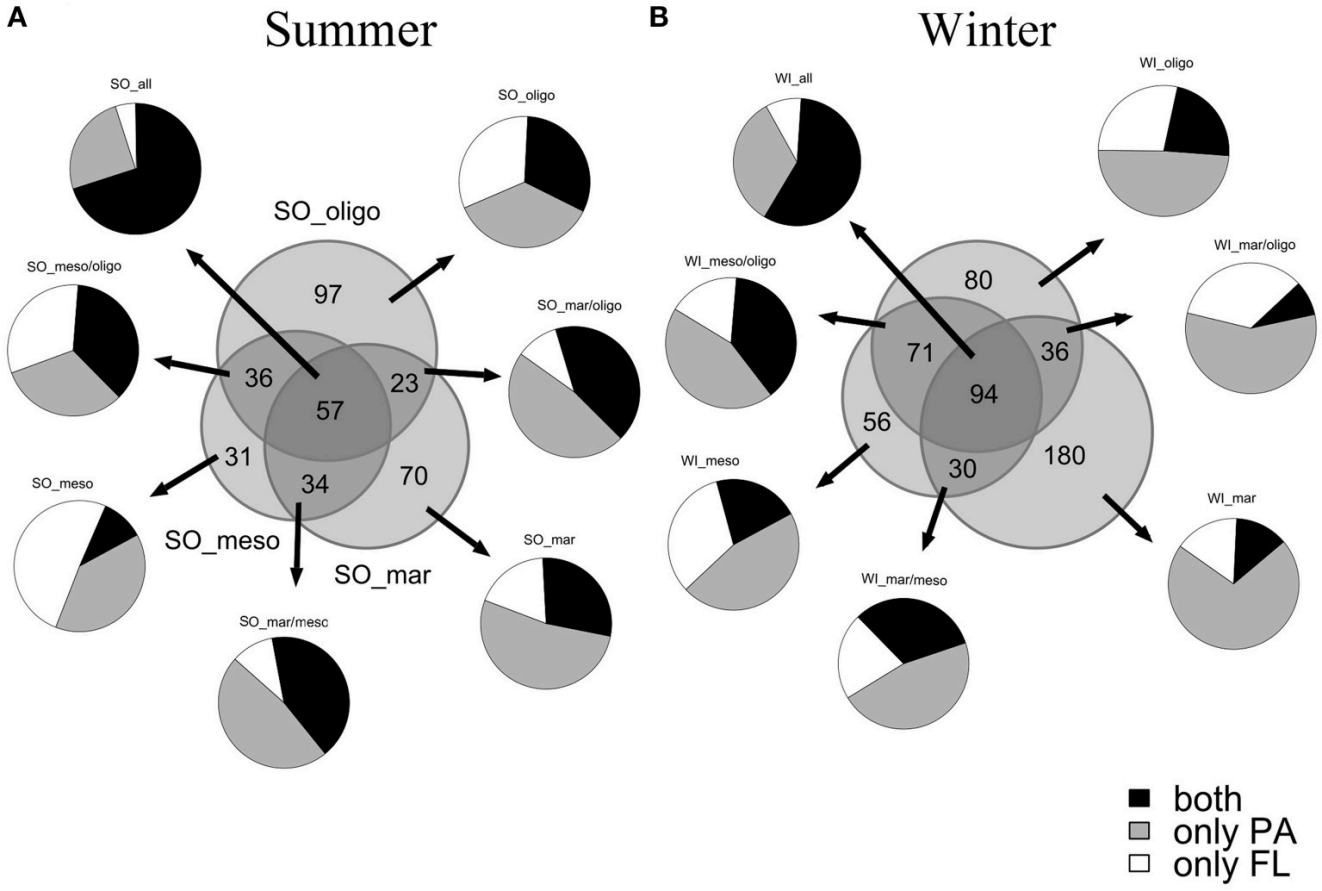
**Venn diagram of OTU distribution of the normalized data between marine (mar), mesohaline (meso) and oligohaline (oligo) samples in summer (A) and winter (B) (areas are proportional to OTU number)**. Relative abundances of all exclusive and shared OTUs in either particle-associated fraction, free-living fraction or both fractions are shown.

**Figure 3:**
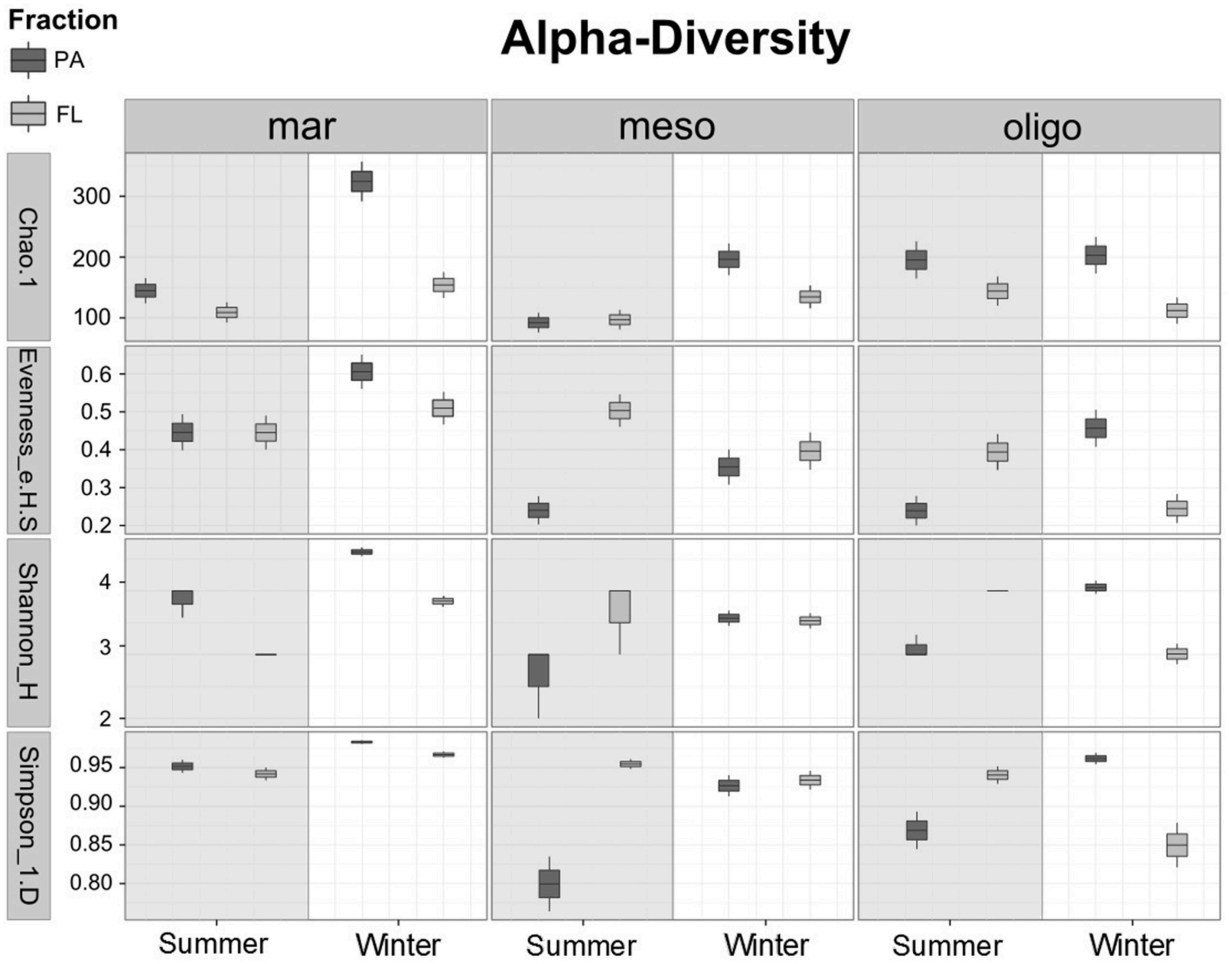
**Boxplots of different α-diversity indices (Chao1, Eveness, Shannon, Simpson) of a randomized dataset (430 reads) to compare the diversity between particle-associated (PA) and free-living (FL) bacteria in summer and winter**. Samples considered as mar: marine, meso: mesohaline and oligo: oligohaline.

The α-diversity measures the OTU diversity of individual samples. The OTU distribution (Figure 2) showed a higher overall bacterial diversity in fall/winter than in summer. Out of the total 348 bacterial OTUs in summer, 184 OTUs were found at the marine, 158 OTUs at the mesohaline and 213 OTUs at the oligohaline stations. In summer, the oligohaline station with the lowest water temperature had the highest total bacterial α-diversity and the mesohaline station the lowest. In fall/winter, the total number of OTUs was higher (547), with 340 OTUs at the marine, 251 OTUs at the mesohaline, and 281 OTUs at the oligohaline station. The majority of OTUs at the mesohaline station was shared with those found at the other stations. Only a small proportion exclusively occurred at the mesohaline station, with a higher proportion in fall/winter. At the mesohaline station, there was a high number of shared OTUs with either the marine or oligohaline stations. Interestingly, when comparing all bacterial fractions, high numbers of both PA and FL OTUs were related to those OTUs, which occurred at all three stations. A high number of exclusively PA OTUs occurred in almost all samples (52% of total OTUs and 59% of the OTUs in winter), with a particularly high number of exclusively PA OTUs at the marine station in both seasons (71% in winter and 53% in summer). A high number of both PA and FL OTUs were shared between oligohaline and mesohaline stations and surprisingly also between the marine and oligohaline stations. Comparison of α-diversity indices (Figure 3) showed that richness was significantly higher in the PA fraction (Wilcoxon rank sum test: *W* = 268, *p* = 0.0008), but a pairwise comparison of each station showed no significant difference for the mesohaline station in both seasons and the oligohaline station in summer (Supplemental Table S1). The “Simpson index” [1-D] indicated that PA and FL fractions were in general evenly distributed at the marine station, but not at the meso- and oligohaline stations, where single taxa dominated the system. “Shannon and Simpson indices” showed higher diversity of PA bacteria at the marine station in both seasons and at the oligohaline station in fall/winter. Evenness was higher in the FL fraction at the mesohaline station in both seasons and the oligohaline station in summer. Generally, samples with a low evenness showed also a low diversity in the Shannon and Simpson indices (raw data shown in Supplemental Table S2).

**Figure 4:**
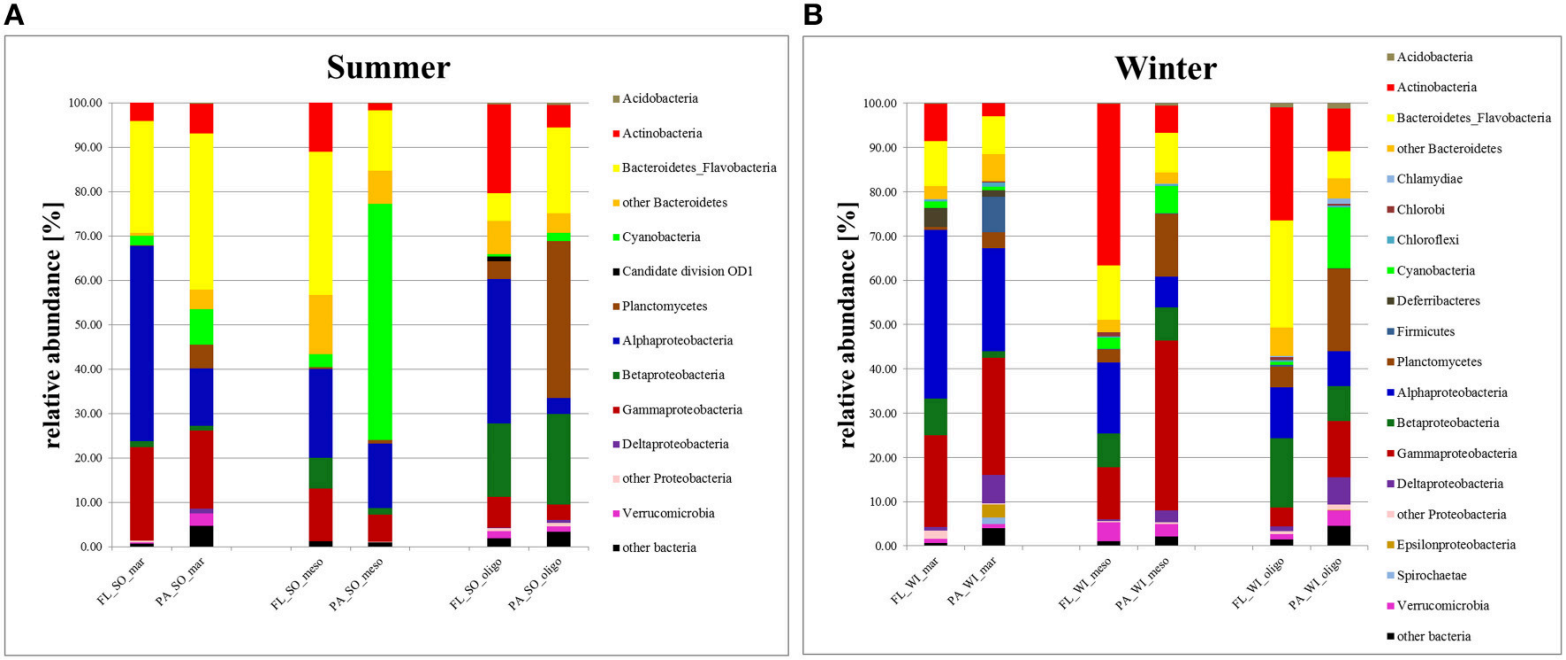
**Taxonomy Plot**. Cumulative bar charts comparing relative class abundances in particle-attached (PA) and free-living (FL) bacteria in samples of summer **(A)** and winter **(B)**.

The β-diversity indices measure the similarity (or dissimilarity) in microbiome composition between samples. At all stations, bacterial communities in Baltic surface waters revealed a higher OTU richness in fall/winter than in summer (Figure 3). The dominant bacterial communities (≥1% of total bacteria) (Figure 4) were *Alpha*−, *Beta*−, *Gamma*−, and *Deltaproteobacteria* as well as *Bacteriodetes*. Also *Flavobacteria, Actinobacteria, Planctomycetes, Cyaonobacteria*, and *Verrucomicrobia* occurred at all stations. *Deferribacteres, Firmicutes*, and *Spirochaetae* were present only at the marine station, whereas *Chloroflexi* were found at the marine and oligohaline station. *Chlorobi* were detected at the meso- and oligohaline stations. *Acidobacteria, Chlamydiae*, and *Candidate division OD1* were exclusively found at the oligohaline station in fall/winter. Richness was lower in summer than in fall/winter since *Firmicutes, Deferribacteres, Chloroflexi, Spirochaetae*, and *Chlorobi* did not occur and *Deltaproteobacteria* and *Verrucomicrobia* were less abundant at the mesohaline station. Only the oligohaline station revealed no considerable difference between winter and summer.

PA bacteria at the marine station were dominated by *Bacteroidetes*, mainly *Flavobacteria*, followed by *Alpha*−, *Beta*−, *Gammaproteobacteria*, and *Cyanobacteria* in summer (Figure 4). In fall/winter, however, bacterial phyla were more evenly distributed among PA bacteria and PA and FL fractions were more similar. FL bacteria were greatly dominated by *Alphaproteobacteria*, followed by *Bacteroidetes* and *Gammaproteobacteria*. *Betaproteobacteria* and *Cyanobacteria* contributed little to FL bacteria. Mesohaline PA bacteria were greatly dominated by *Cyanobacteria*, but otherwise they were relatively similar to FL bacteria with *Bacteroidetes, Alpha*-, and *Gammaproteobacteria* being the major bacterial groups. In fall/winter, the meso- and oligohaline samples comprised a relatively high number of *Actinobacteria* in the FL fraction, whereas the PA bacteria showed higher numbers of *Gammaproteobacteria* and *Planctomycetes* compared to the FL fraction.

PA bacteria at the oligohaline station were dominated by *Planctomycetes* in summer (Figure 4), followed by *Bacteroidetes* and *Betaproteobacteria*. In contrast, the FL fraction was characterized by a dominance of *Alpha*− and *Betaproteobacteria* as well as *Actinobacteria*. In fall/winter, the oligohaline FL BCC was characterized by a high abundance of *Actinobacteria*, and a higher amount of *Planctomycetes, Gammaproteobacteria*, and *Cyanobacteria* compared to the PA fraction. Except for the high amount of *Alphaproteobacteria* at the oligohaline station in summer, the relative abundance of *Alphaproteobacteria* and *Gammaproteobacteria* increased with salinity, whereas *Actinobacteria* and *Betaproteobacteria* decreased. For PA bacteria there was a trend of increasing dominance of *Bacteroidetes* from higher to lower salinity levels, whereas *Planctomycetes* showed the opposite trend.

**Figure 5:**
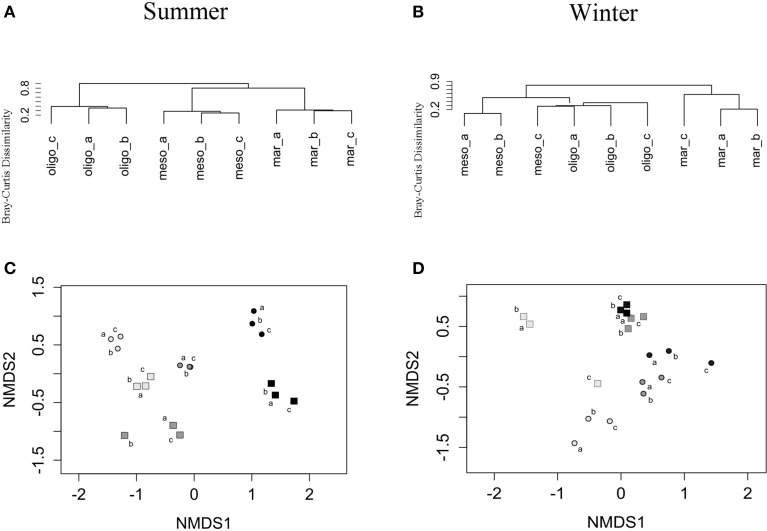
**Unweighted-pair group method with arithmetic mean (UPGMA) dendrograms based on Bray–Curtis dissimilarities of total bacteria in summer (A) and winter samples (B) of the three stations (marine, mesohaline, oligohaline)**. And non-metric multidimensional scaling (NMDS) for particle-associated (PA) and free-living (FL) bacteria in summer **(C)** and winter samples **(D)** of the three stations, with light gray: stations considered as “marine” (mar), dark gray: stations considered as “mesohaline” (meso) and black: stations considered as “oligohaline” (oligo). So, summer; Wi, winter; squares, PA; and dots, FL.

The total BCC revealed significant differences between marine, mesohaline and oligohaline stations for both studied seasons (Table 2) even though this shift was less clear in fall/winter. In summer, BCC of the mesohaline station was more similar to the marine station, whereas in fall/winter it was more similar to the oligohaline station (Figures 5A,B). BCC varied with stations, fractions, and sampling time, except BCC of the oligohaline station did not significantly differ between both seasons (Table 2). Supplemental Figure S1C shows that this pattern was mainly explained by FL bacteria being highly similar in summer and fall/winter. Changes in BCC with stations revealed a stronger significance in summer (*R* = 1.00, *p* = 0.002) than in fall/winter (*R* = 0.78, *p* = 0.003), and differences in BCC between stations were lower when comparing the neighboring stations (mesohaline vs. marine or mesohaline vs. oligohaline) (Table 2). Differences in BCC were mainly explained by the higher overall bacterial α-diversity in fall/winter compared to the summer samples and changes in the dominant phyla (Figure 3). Differences between PA and FL BCC were more significant in fall/winter than in summer (Table 2). Thereby, the mesohaline station showed, together with the oligohaline station, the lowest differences between FL and PA fractions in summer (Supplemental Figures S1A–D). At all stations there were differences in PA vs. FL BCC in summer as well as in fall/winter (Figures 5C,D). The high BCC similarity of meso- and oligohaline samples in fall/winter is based on both fractions (PA and FL). Generally, there was a significant difference between PA and FL BCC within stations or seasons (Table 2, Supplemental Figure S2). Comparing each sample within season and station with a Wilcoxon rank sum test revealed a significant difference between both fractions at the marine station in both seasons as well as at the oligohaline station in fall/winter. Differences between bacteria fractions at the mesohaline station, however, remained non-significant (Supplemental Table S1).

**Table 2:** **Results of Analysis of Similarities (ANOSIM) to test for significant differences in bacterial community composition (BCC) between stations (marine, mesohaline, oligohaline), fractions [particle-associated (PA) vs. free-living (FL)], and seasons (summer vs. fall/winter)**.

**Sample**	**N**	**Factor**	***R***	***p*-value**	**Significance**
All	18	Stations	0.73	0.001	^***^
Summer	9	Stations	1.00	0.002	^**^
Fall/winter	9	Stations	0.78	0.003	^**^
Mar/meso	12	Stations	0.61	0.007	^**^
Mar/oligo	12	Stations	1	0.003	^**^
Meso/oligo	12	Stations	0.53	0.002	^**^
Summer	9	Fractions	0.19	0.035	^*^
Fall/winter	9	Fractions	0.41	0.001	^***^
Marine	6	Fractions	0.46	0.001	^***^
Mesohaline	6	Fractions	0.32	0.046	^*^
Oligohaline	6	Fractions	0.76	0.002	^**^
Marine	6	Seasons	0.36	0.011	^*^
Mesohaline	6	Seasons	0.90	0.001	^***^
Oligohaline	6	Seasons	ns	ns	ns

For stations, BCC of total bacteria was tested. For fractions and seasons, BCC of PA and FL bacteria were tested separately. R, explained variance. p-value, level of significance and ns, not significant (

* *p < 0.05*,

** *p < 0.01*,

*** *p ≤ 0.001)*.

## Discussion

Particles such as phytoplankton and terrestrial POM provide important habitats for bacteria (e.g., Grossart, 2010) and drive bacterial diversity and dynamics (e.g., Rooney-Varga et al., 2005). Many studies indicate pronounced differences in community composition of PA and FL bacteria at various sampling sites (Acinas et al., 1999; Crump et al., 1999; Ortega-Retuerta et al., 2013; Bižić-Ionescu et al., 2014). Yet, it remains unclear whether PA and FL bacteria show systematic differences in diversity and dynamics when environmental conditions, in particular salinity and DOC, change (Smith et al., 2013). Therefore, we have set a particular focus on the comparison of PA vs. FL bacterial communities at 3 sampling stations with different environmental conditions during two contrasting seasons: summer vs. fall/winter. The Baltic Sea provides an ideal model system for such studies since it is characterized by strong and relatively stable gradients in salinity and DOC from north to south resulting in decreasing amounts of organic matter and nutrients. Our study revealed that PA OTUs account for 52% of overall total OTUs and thus represent an important component of bacterial α-diversity. Dynamics of PA and FL BCC differed in relation to environmental settings, in particular those related to phytoplankton bloom development. Whereas relative OTU and absolute cell abundance of PA bacteria was low (< 10%), their significantly higher csBPP rates than FL bacteria indicates that PA bacteria represent an important component of Baltic Sea bacterioplankton (Simon et al., 2002, 2014; Grossart, 2010).

### Bacterial communities in relation to changes in environmental variables

The first sampling cruise was performed during November/December 2011 to account for the fall/winter season. Autumn 2011, however, was unusually warm (4–5°C higher than average November temperatures during the past 20 years) and the first ice cover of the Bothnian Bay was delayed by ca. 2 weeks (8th of December). Yet, we assigned this sampling period as “fall/winter,” since it was characterized by an accumulation of nutrients, short daylight, and a relatively low phytoplankton biomass. In contrast, our “summer” sampling was performed during May/June 2012 when nutrient concentrations were already low, daylight periods long, and a bloom of cyanobacteria and diatoms occurred (Nausch et al., 2013). However, due to relatively unfavorable weather conditions (cold and windy) the phytoplankton bloom was not as intense as in the years before. Moreover, in 2012, due to a relatively cold summer and absence of the normal temperature rise in May/June water temperatures differed little between seasons and stations. The oligohaline station differed from the other stations by low temperature and bacterial activities as well as a relatively high nitrate concentration in summer, due to its ice coverage until mid-May (Nausch et al., 2013). The phytoplankton bloom development at the mesohaline station in summer can serve as an explanation for the observed high abundance and proportion of PA bacteria.

BCC and bacterial α-diversity differed with salinity and between both sampling periods with generally higher species richness in fall/winter. It is known that bacterial species richness generally decreases during bloom situations in summer (Hodges et al., 2005). This is supported by recent studies in coastal surface waters which indicate the highest bacterial richness during winter (Gilbert et al., 2010; Ghiglione and Murray, 2012). In the Baltic Sea, a larger resource heterogeneity can be also expected in fall/winter when phytoplankton blooms are relatively rare, whereas resources during phytoplankton blooms, particularly in summer, seem to be more homogeneous and may lead to the observed reduction in bacterial richness in summer (Crespo et al., 2013). Differences between PA and FL BCC were also more significant in fall/winter, when precipitation and hence terrestrial runoff of POM were higher than in summer. Changes in water conditions and concentrations of particulate matter (e.g., POC, PN) as well as dissolved variables (e.g., DOC) were shown to correlate with seasonal changes in abundant taxa of heterotrophic bacteria (Crump et al., 2003; Fortunato et al., 2013). Recently, differences in the lifestyles and metabolic capabilities of PA and FL bacterial fractions were found by metagenome studies in coastal as well as open ocean areas (Simon et al., 2014; Fontanez et al., 2015).

We found a dominance of the typical bacterial community in surface waters of the Baltic Sea (Herlemann et al., 2011). The number of exclusively mesohaline taxa in the PA fraction was low in summer and accounts for only half of the taxa occurring at the two other stations. In contrast, number of FL OTUs was higher at the mesohaline station than at the marine one. In fall/winter, PA fractions at the meso- and oligohaline stations were less diverse than at the marine station, whereas FL bacteria showed no distinct differences. The more pronounced differences between BCC of FL and PA bacterial fractions in fall/winter than in summer could be explained by changes in composition and hence particle quality (>5 μm) throughout the season. Differences between PA and FL bacteria are not necessarily correlated to particle concentration, but to SPM quality (Doxaran et al., 2012). Generally, PA bacteria have larger genome sizes than FL (Allen et al., 2012), and especially PA bacteria contain more transporters that can be linked to the successive decomposition of phytoplankton blooms (Teeling et al., 2012; Smith et al., 2013). This enables a flexible and rapid adaptation to changes in various environmental conditions, e.g., snow melt or phytoplankton blooms, by switching between different life-styles.

A substantial fraction (52%) of all retrieved OTUs occurred exclusively in the PA fraction of all samples. In contrast, numbers of exclusively FL OTUs were often lower than for the PA fraction indicating that particles (>5 μm) serve as important hotspots for bacterial α-diversity (Simon et al., 2002; Grossart, 2010; Rösel and Grossart, 2012; Bižić-Ionescu et al., 2014) also in the Baltic Sea. This notion is supported by the generally higher bacterial richness of PA than FL bacteria, except for the mesohaline station in summer. The increase in number of PA bacteria in summer at the marine and mesohaline stations were related to phytoplankton development. In contrast, at the relatively cold oligohaline station number of PA bacteria remained at the same high level in both seasons indicating that PA bacteria greatly benefit from riverine terrestrial input at this station, in particular in fall/winter when riverine freshwater runoff is high. This notion is supported by the higher proportion of PA bacteria at the oligohaline station in fall/winter, whereas the mesohaline station showed the opposite trend.

Since the mesohaline site contains only a small number of exclusively mesohaline taxa (ca. 20%) the mesohaline site may represent a mixture of oligohaline and marine bacteria. Herlemann et al. (2011) found that the brackish waters of the Baltic Sea are occupied by a diverse combination of freshwater and marine clades that seem to have adapted to the brackish conditions. Consequently, α- diversity changed little at the mesohaline station between both seasons.

Bacterial richness was highest at the marine station in fall/winter with 71% of all marine OTUs, and 59% of total OTUs in winter samples exclusively occurred in the PA fraction which suggests that PA bacteria are generally more diverse than FL bacteria. Several studies have shown that PA bacteria greatly contribute to the overall bacterial diversity in surface waters (LaMontagne and Holden, 2003; Crespo et al., 2013; Ortega-Retuerta et al., 2013; Bižić-Ionescu et al., 2014). For the Northwest Mediterranean, Sea Crespo et al. (2013) showed that almost half of all OTUs (49%) exclusively occur in the PA fraction, 25% were shared and 26% exclusively occur in the FL fraction. The authors have also shown that OTUs which stem exclusively from PA or FL bacteria were in general very low in abundance (6% of total abundance). Whereas most of the previous studies indicate a higher richness of FL compared to PA bacteria (Acinas et al., 1999; Hollibaugh et al., 2000; Ghiglione et al., 2009; Kellogg and Deming, 2009), our study indicates a higher richness of PA bacteria in almost all samples. This finding is supported by metagenome studies specifically differentiating between PA and FL fractions in samples from a coastal margin (Simon et al., 2014). A possible reason for this discrepancy can be related to the fact that previous studies, without next generation sequencing, may have analyzed only the most abundant taxa and hence missed a large portion of total bacterial α-diversity. The 454-pyrosequencing approach has been successfully used to estimate also taxa of low abundance (Sogin et al., 2006; Pedrós-Alió, 2012) leading to a much higher bacterial α-diversity and richness, in particular of PA bacteria. Despite this advantage it should be kept in mind that sequencing errors of pyro-sequencing can bias the true number of rare taxa. Since we have quality-filtered our sequences according to Kunin et al. (2010) including removal of a) reads with one or more unresolved bases (Ns), b) errors in the barcode or primer sequence and c) atypically short or long reads as well as using d) a clustering threshold not greater than 97% identity, we think that an overestimation of OUT richness is unlikeley. It should be also mentioned that the total amount of reads per sample can vary by orders of one magnitude within a single sequencing run. Randomly subsampling the same number of reads for each sample based on the smallest sample size is a common approach to allow for direct comparisons of samples (Gilbert et al., 2010; Crespo et al., 2013; Ortega-Retuerta et al., 2013). To avoid false interpretations in diversity of samples with very low reads, a rarefaction curve was performed to allow the calculation of species richness for a given number of individual samples. Though the rarefaction curves did not reach an asymptote, the most underestimated samples are the ones with already the highest diversity (Supplemental Figure S3). The function “rrarefy” generates randomly rarefied community data without replacement, so that the variance of rarefied communities is rather related to rarefaction proportion than to the sample size.

### Specific phylogenetic findings

Similar to Herlemann et al. (2011), our observations revealed a higher abundance of *Gammaproteobacteria* with increasing salinity and the opposite trend for *Actinoacteria* and *Betaproteobacteria* (also see Bižić-Ionescu et al., 2014). Contrary to earlier observations (Herlemann et al., 2011) indicating a decreasing abundance of *Alphaproteobacteria* (mainly *Pelagibacterales*) at lower salinities, we detected a surprisingly high abundance of FL *Alphaproteobacteria* at the oligohaline station in summer. The predominant taxa at the oligohaline station belonged to the *Pelagibacterales* previously found in Chesapeake Bay. Chesapeake Bay is the largest estuary in the United States. Kan and colleagues have shown that the *Pelagibacterales* (SAR11-related) clones appeared in the Chesapeake Bay and the Delaware Estuary only in the warm season (Kan et al., 2007), which correlates with our findings.

The phylogenetic distribution of bacteria at both sampling time points is in accordance with observations of Andersson et al. (2009), who analyzed the seasonal pattern of BCC (from May to October 2008) at the Landsort Deep. In this study, dynamics of *Alpha*− and *Beta-Proteobacteria* were closely related to a cyanobacterial bloom. *Bacteroidetes* showed a peak in summer (June) and a decrease in fall (October) when both organic matter concentrations and temperatures dropped. A similar temporal development was also found for *Actinobacteria* which were more dominant in late July until October. Contrary to our results, Herlemann et al. (2011) described a large fraction of *Verrucomicrobia* in summer (July) at the mesohaline site. This discrepancy might be related to differences in sampling years and time points. According to Andersson et al. (2009), the peak of *Verrucomicrobia* occurred from late June to August which is later than our summer and earlier than our fall/winter sampling. Therefore, it is very likely that we have missed this *Verrucomicrobia* peak with our sampling scheme. In fall/winter, the mesohaline station showed a relatively high number of *Actinomybacteria* in the FL fraction and a dominance of *Gammaproteobacteria* and *Planctomycetes* in the PA fraction, which indicates the higher presence of oligohaline bacteria in fall/winter, probably due to higher riverine freshwater runoff and reduced phytoplankton influence.

In our study, there was a high proportion of PA *Planctomycetes* and *Flavobacteria* which greatly differ in their life-style. Interestingly, we found a dominance of PA *Planctomycetes* exclusively in the oligohaline samples (35% of PA bacteria in summer and 19% in fall/winter), which mainly consisted of CL500-3 (95% of PA *Planctomycetes*). CL500-3 was also the most significant driver of the separation between fractions (SIMPER: 5%) and between the separation of the oligohaline and the mesohaline as well as the marine station (SIMPER: 7 and 6%). This group is typically found in freshwater and was first described in an oligotrophic crater lake (Urbach et al., 2001). They tend to a PA lifestyle and thus drive the separation of BCC's in different habitats (Jackson et al., 2014). *Planctomycetes* seem to be involved in the mineralization of algal biomass, which is supported by the positive correlation between their abundance and chl*a* concentrations (Pizzetti et al., 2011). *Bacteriodetes-Flavobacteria* were less abundant in these samples, but dominated at the marine station. *Flavobacteria* occur in marine and freshwater habitats as typical PA bacteria since they represent metabolic generalists with a broad range of polysaccharide degrading enzymes (Jeske et al., 2013; Walsh et al., 2013). The marine station in summer was dominated by *Polaribacter, Fluviicola* and *Formosa* genera; both *Polaribacter* and *Fluviicola* were previously found on heterogenous particles (Bižić-Ionescu et al., 2014). *Formosa* was identified as an early colonizer of a decaying diatom bloom (Teeling et al., 2012). *Flavobacterium* was mainly found in the oligohaline and mesohaline stations, but seemed to be quite stable in both seasons (around 4%) at the oligohaline station. However, this group was an important member in structuring the fractions (SIMPER 4%).

In conclusion, strong differences in BCC as well as in α− and β-diversity occurred among stations with different salinities and seasons revealing significantly higher species richness in fall/winter than in summer. PA bacteria comprise a large number of less abundant OTUs, especially at the oligotrophic marine site. At the marine station, α-diversity of PA bacteria was in both seasons higher than of FL bacteria and substantially contributed to overall bacterial diversity. Interestingly, key PA bacteria consisted of *Planctomycetes* at the oligohaline station and of *Flavobacteria* at the marine station, which differ greatly in their life-style. PA bacteria—although usually less abundant (<10% of total bacteria)—were characterized by higher cell specific activities and consequently greatly contribute to overall BPP. Thus, PA bacteria represent an important component of overall bacterial α- and β-diversity and presumably activity in the Baltic Sea.

### Conflict of interest statement

The authors declare that the research was conducted in the absence of any commercial or financial relationships that could be construed as a potential conflict of interest.

## Supplementary material

Supplemental Figure S1 **Unweighted-pair group method with arithmetic mean (UPGMA) dendograms based on Bray-Curtis dissimilarities of PA and FL bacteria distribution at different stations [marine (A), mesohaline (B), oligohaline (C), and for all samples (D)]**.

Supplemental Figure S2 **Unweighted-pair group method with arithmetic mean (UPGMA) dendograms based on Bray-Curtis dissimilarities of the distribution of PA and FL bacteria at different stations in summer and winter marine (A,D), mesohaline (B,E), oligohaline (C,F)**.

Supplemental Figure S3 **Rarefaction curves for free-living and particle-associated samples of the total dataset**.

Supplemental Table S1 **Wilcoxon Rank sum test between all samples within station and season and a factor (F) from 0 to 1, which compares the groups [triplicates (a, b, c) vs. fractions (PA, FL)] within one station (mar, meso, oligo) and season (summer, fall/winter) to estimate the difference between the fractions, with 1 as highest possible difference**.

Supplemental Table S2 **Number of OTUs, reads, Chao1, Shannon [H], and Simpson [1-D] and Buzas and Gibson's Evenness [e^∧^H/S] diversity indexes of averages of samples after normalization to 430 reads per sample**. PA, particle-attached; FL, free-living; PA, particle-associated fraction; FL, free-living fraction; Tot, total bacteria.

## References

[B1] Abràmoff M. D. Magalhães P. J. Ram S. J. (2004). Image processing with ImageJ. Biophotonics Int. 11, 36–43. Available online at: http://dspace.library.uu.nl/handle/1874/204900

[B2] Acinas S. G. Antón J. Rodríguez-Valera F. (1999). Diversity of free-living and attached bacteria in offshore western Mediterranean waters as depicted by analysis of genes encoding 16S rRNA. Appl. Environ. Microbiol. 65, 514–522. 9925576 10.1128/aem.65.2.514-522.1999 PMC91055

[B3] Alldredge A. L. Silver M. W. (1988). Characteristics, dynamics and significance of marine snow. Prog. Oceanogr. 20, 41–82. 10.1016/0079-6611(88)90053-5

[B4] Allen L. Z. Allen E. E. Badger J. H. McCrow J. P. Paulsen I. T. Elbourne L. D. H.. (2012). Influence of nutrients and currents on the genomic composition of microbes across an upwelling mosaic. ISME J. 6, 1403–1414. 10.1038/ismej.2011.201 22278668 PMC3379637

[B5] Andersson A. F. Riemann L. Bertilsson S. (2009). Pyrosequencing reveals contrasting seasonal dynamics of taxa within Baltic Sea bacterioplankton communities. ISME J. 4, 171–181. 10.1038/ismej.2009.108 19829318

[B6] Bižić-Ionescu M. Zeder M. Ionescu D. Orlić S. Fuchs B. M. Grossart H.-P.. (2014). Comparison of bacterial communities on limnic versus coastal marine particles reveals profound differences in colonization. Environ. Microbiol. 17, 3500–3514. 10.1111/1462-2920.12466 24674021

[B7] Buesing N. (2005). Bacterial counts and biomass determination by epifluorescence microscopy, in Methods to Study Litter Decomposition SE - 27, eds Graça M. S. Bärlocher F. Gessner M. (Dordrecht: Springer), 203–208.

[B8] Crespo B. G. Pommier T. Fernández−Gómez B. Pedrós−Alió C. (2013). Taxonomic composition of the particle−attached and free−living bacterial assemblages in the Northwest Mediterranean Sea analyzed by pyrosequencing of the 16S rRNA. Microbiologyopen 2, 541–552. 10.1002/mbo3.92 23723056 PMC3948605

[B9] Crump B. C. Armbrust E. V. Baross J. A. (1999). Phylogenetic analysis of particle-attached and free-living bacterial communities in the Columbia River, its estuary, and the adjacent coastal ocean. Appl. Environ. Microbiol. 65, 3192–3204. 10388721 10.1128/aem.65.7.3192-3204.1999 PMC91474

[B10] Crump B. C. Baross J. A. Simenstad C. A. (1998). Dominance of particle-attached bacteria in the Columbia River estuary, USA. Aquat. Microb. Ecol. 14, 7–18. 10.3354/ame014007

[B11] Crump B. C. Hopkinson C. S. Sogin M. L. Hobbie J. E. (2004). Microbial biogeography along an estuarine salinity gradient: combined influences of bacterial growth and residence time. Appl. Environ. Microbiol. 70, 1494–1505. 10.1128/AEM.70.3.1494-1505.2004 15006771 PMC365029

[B12] Crump B. C. Kling G. W. Bahr M. Hobbie J. E. (2003). Bacterioplankton community shifts in an arctic lake correlate with seasonal changes in organic matter source. Appl. Environ. Microbiol. 69, 2253–2268. 10.1128/AEM.69.4.2253-2268.2003 12676708 PMC154827

[B13] Doxaran D. Ehn J. Bélanger S. Matsuoka A. Hooker S. Babin M. (2012). Optical characterisation of suspended particles in the Mackenzie River plume (Canadian Arctic Ocean) and implications for ocean colour remote sensing. Biogeosciences 9, 3213–3229. 10.5194/bg-9-3213-2012

[B14] Edwards L. E. John Pojeta J. (1997). Fossils, Rocks, and Time. Available online at: http://pubs.usgs.gov/gip/fossils/contents.html

[B15] Fogg P. G. T. Gerrard W. (1990). Solubility of Gases in Liquids. Chichester: John Wiley.

[B16] Fontanez K. M. Eppley J. M. Samo T. J. Karl D. M. DeLong E. F. (2015). Microbial community structure and function on sinking particles in the North Pacific Subtropical Gyre. Front. Microbiol. 6:469. 10.3389/fmicb.2015.00469 26042105 PMC4436931

[B17] Fortunato C. S. Eiler A. Herfort L. Needoba J. A. Peterson T. D. Crump B. C. (2013). Determining indicator taxa across spatial and seasonal gradients in the Columbia River coastal margin. ISME J. 7, 1899–1911. 10.1038/ismej.2013.79 23719153 PMC3965310

[B18] Fowler S. W. Knauer G. A. (1986). Role of large particles in the transport of elements and organic compounds through the oceanic water column. Prog. Oceanogr. 16, 147–194. 10.1016/0079-6611(86)90032-7

[B19] Ganesh S. Bristow L. A. Larsen M. Sarode N. Thamdrup B. Stewart F. J. (2015). Size-fraction partitioning of community gene transcription and nitrogen metabolism in a marine oxygen minimum zone. ISME J. 9, 2682–2696. 10.1038/ismej.2015.44 25848875 PMC4817638

[B20] Garneau M.-È. Vincent W. F. Terrado R. Lovejoy C. (2009). Importance of particle-associated bacterial heterotrophy in a coastal Arctic ecosystem. J. Mar. Syst. 75, 185–197. 10.1016/j.jmarsys.2008.09.002

[B21] Ghiglione J.-F. Conan P. Pujo-Pay M. (2009). Diversity of total and active free-living vs. particle-attached bacteria in the euphotic zone of the NW Mediterranean Sea. FEMS Microbiol. Lett. 299, 9–21. 10.1111/j.1574-6968.2009.01694.x 19686348

[B22] Ghiglione J. F. Mevel G. Pujo-Pay M. Mousseau L. Lebaron P. Goutx M. (2007). Diel and seasonal variations in abundance, activity, and community structure of particle-attached and free-living bacteria in NW mediterranean sea. Microb. Ecol. 54, 217–231. 10.1007/s00248-006-9189-7 17345139

[B23] Ghiglione J. F. Murray A. E. (2012). Pronounced summer to winter differences and higher wintertime richness in coastal Antarctic marine bacterioplankton. Environ. Microbiol. 14, 617–629. 10.1111/j.1462-2920.2011.02601.x 22003839

[B24] Gilbert J. A. Field D. Swift P. Thomas S. Cummings D. Temperton B.. (2010). The taxonomic and functional diversity of microbes at a temperate coastal site: a “multi-omic”study of seasonal and diel temporal variation. PLoS ONE 5:e15545. 10.1371/journal.pone.0015545 21124740 PMC2993967

[B25] Grasshoff K. Ehrhardt M. Kremling K. (1983). Methods of Seawater Analyses. Weinheim: Verlag Chemie.

[B26] Grossart H. P. (2010). Ecological consequences of bacterioplankton lifestyles: changes in concepts are needed. Environ. Microbiol. Rep. 2, 706–714. 10.1111/j.1758-2229.2010.00179.x 23766274

[B27] Grossart H. P. Levold F. Allgaier M. Simon M. Brinkhoff T. (2005). Marine diatom species harbour distinct bacterial communities. Environ. Microbiol. 7, 860–873. 10.1111/j.1462-2920.2005.00759.x 15892705

[B28] Grossart H. P. Simon M. (1993). Limnetic macroscopic organic aggregates (Lake Snow): occurrence, characteristics, and microbial dynamics in lake constance. Limnol. Oceanogr. 38, 532–546. 10.4319/lo.1993.38.3.0532

[B29] Grossart H. P. Tang K. W. Turk V. (2010). Linkage between crustacean zooplankton and aquatic bacteria. Aquat. Microb. Ecol. 61, 261–277. 10.3354/ame01424

[B30] Hammer O. Harper D. A. T. Ryan P. D. (2012). PAST: paleontological statistics software package for education and data analysis. Paleontol Electron 4 (art. 4):9 Available online at: http://palaeo-electronica.org/2001_1/past/issue1_01.htm

[B31] Herlemann D. P. R. Labrenz M. Jürgens K. Bertilsson S. Waniek J. J. Andersson A. F. (2011). Transitions in bacterial communities along the 2000[thinsp]km salinity gradient of the Baltic Sea. ISME J. 5, 1571–1579. 10.1038/ismej.2011.41 21472016 PMC3176514

[B32] Hodges L. R. Bano N. Hollibaugh J. T. Yager P. L. (2005). Illustrating the importance of particulate organic matter to pelagic microbial abundance and community structure-an Arctic case study. Aquat. Microb. Ecol. 40, 217–227. 10.3354/ame040217

[B33] Hollibaugh J. T. Wong P. S. Murrell M. C. (2000). Similarity of particle-associated and free-living bacterial communities in northern San Francisco Bay, California. Aquat. Microb. Ecol. 21, 103–114. 10.3354/ame021103

[B34] Holmfeldt K. Dziallas C. Titelman J. Pohlmann K. Grossart H. P. Riemann L. (2009). Diversity and abundance of freshwater Actinobacteria along environmental gradients in the brackish northern Baltic Sea. Environ. Microbiol. 11, 2042–2054. 10.1111/j.1462-2920.2009.01925.x 19453610

[B35] Ionescu D. Siebert C. Polerecky L. Munwes Y. Y. Lott C. Häusler S.. (2012). Microbial and chemical characterization of underwater fresh water springs in the Dead Sea. PLoS ONE 7:e38319. 10.1371/journal.pone.0038319 22679498 PMC3367964

[B36] Jackson C. R. Millar J. J. Payne J. T. Ochs C. A. (2014). Free-living and particle-associated bacterioplankton in large rivers of the Mississippi River Basin demonstrate biogeographic patterns. Appl. Environ. Microbiol. 80, 7186–7195. 10.1128/AEM.01844-14 25217018 PMC4249191

[B37] Jeske O. Jogler M. Petersen J. Sikorski J. Jogler C. (2013). From genome mining to phenotypic microarrays: planctomycetes as source for novel bioactive molecules. Antonie Van Leeuwenhoek 104, 551–567. 10.1007/s10482-013-0007-1 23982431

[B38] Kan J. Suzuki M. T. Wang K. Evans S. E. Chen F. (2007). High temporal but low spatial heterogeneity of bacterioplankton in the Chesapeake Bay. Appl. Environ. Microbiol. 73, 6776–6789. 10.1128/AEM.00541-07 17827310 PMC2074944

[B39] Kautsky L. Kautsky N. (2000). The baltic sea, including bothnian sea and bothnian bay, in Seas at the Millennium: An Environmental Evaluation: 1. Regional Chapters: Europe, The Americas and West Africa, ed Sheppard C. R. C. (Amsterdam: Pergamon), 121–133.

[B40] Kellogg C. T. E. Deming J. W. (2009). Comparison of free-living, suspended particle, and aggregate-associated bacterial and archaeal communities in the Laptev Sea. Aquat. Microb. Ecol. 57, 1–18. 10.3354/ame01317

[B41] Klindworth A. Pruesse E. Schweer T. Peplies J. Quast C. Horn M.. (2013). Evaluation of general 16S ribosomal RNA gene PCR primers for classical and next-generation sequencing-based diversity studies. Nucleic Acids Res. 41:e1. 10.1093/nar/gks808 22933715 PMC3592464

[B42] Köster M. Paffenhöfer G.-A. Schlüter R. Meuche A. (2012). Time-series observations of prokaryotic colonization of zooplankton fecal pellets. J. Plankton Res. 36, 1461–1475. 10.1093/plankt/fbu060

[B43] Krebs C. (1989). Ecological Methodology. New York, NY: Harper Collins Publishers.

[B44] Kunin V. Engelbrektson A. Ochman H. Hugenholtz P. (2010). Wrinkles in the rare biosphere: pyrosequencing errors can lead to artificial inflation of diversity estimates. Environ. Microbiol. 12, 118–123. 10.1111/j.1462-2920.2009.02051.x 19725865

[B45] LaMontagne M. G. Holden P. A. (2003). Comparison of free-living and particle-associated bacterial communities in a coastal lagoon. Microb. Ecol. 46, 228–237. 10.1007/s00248-001-1072-y 14708747

[B46] Li W. Godzik A. (2006). Cd-hit: a fast program for clustering and comparing large sets of protein or nucleotide sequences. Bioinformatics 22, 1658–1659. 10.1093/bioinformatics/btl158 16731699

[B47] Lyons M. M. Dobbs F. C. (2012). Differential utilization of carbon substrates by aggregate-associated and water-associated heterotrophic bacterial communities. Hydrobiologia 686, 181–193. 10.1007/s10750-012-1010-7

[B48] Martinez J. Smith D. C. Steward G. F. Azam F. (1996). Variability in ectohydrolytic enzyme activities of pelagic marine bacteria and its significance for substrate processing in the sea. Aquat. Microb. Ecol. 10, 223–230. 10.3354/ame010223

[B49] Massana R. Gasol J. M. Bjørnsen P. K. Blackburn N. Hagstrøm Å. Hietanen S. (1997). Measurement of bacterial size via image analysis of epifluorescence preparations: description of an inexpensive system and solutions to some of the most common problems. Sci. Mar. 61, 397–407.

[B50] Nausch G. Feistel R. Umlauf L. Mohrholz V. Siegel H. (2013). Hydrographisch-hydrochemische Zustandseinschätzung der Ostsee 2012, in Marine Science Reports, Vol. 91, ed Wasmund D. N. (Rostock-Warnemünde: Leibniz Institute for Baltic Sea Research Warnemünde), 1–109. 10.12754/msr-2013-0091

[B51] Nercessian O. Noyes E. Kalyuzhnaya M. G. Lidstrom M. E. Chistoserdova L. (2005). Bacterial populations active in metabolism of C1 compounds in the sediment of Lake Washington, a freshwater lake. Appl. Environ. Microbiol. 71, 6885–6899. 10.1128/AEM.71.11.6885-6899.2005 16269723 PMC1287692

[B52] Oksanen J. Blanchet F. G. Kindt R. Legendre P. Minchin P. R. O'Hara R. B. (2011). Vegan: Community Ecology Package Version 2.0-2. R CRAN Packag.

[B53] Ortega-Retuerta E. Joux F. Jeffrey W. H. Ghiglione J. F. (2013). Spatial variability of particle-attached and free-living bacterial diversity in surface waters from the Mackenzie River to the Beaufort Sea (Canadian Arctic). Biogeosciences 10, 2747–2759. 10.5194/bg-10-2747-2013

[B54] Pedrós-Alió C. (2012). The rare bacterial biosphere. Ann. Rev. Mar. Sci. 4, 449–466. 10.1146/annurev-marine-120710-100948 22457983

[B55] Pizzetti I. Gobet A. Fuchs B. M. Amann R. Fazi S. (2011). Abundance and diversity of Planctomycetes in a Tyrrhenian coastal system of central Italy. Aquat. Microb. Ecol. 65, 129–141. 10.3354/ame01535

[B56] R Core Team (2013). R: A Language and Environment for Statistical Computing. R Foundation for Statistical Computing Available online at: http://www.r-project.org/

[B57] Remane A. (1934). Die *Brackwasserfauna*. Frankfurt: Verhandlungen der Deutschen Zoologischen Gesellschaft.

[B58] Robinson C. Archer S. D. Le B. Williams P. J. (1999). Microbial dynamics in coastal waters of East Antarctica: plankton production and respiration. Mar. Ecol. Prog. Ser. 180, 23–36. 10.3354/meps180023

[B59] Rohde K.-H. Nehring D. (1979). Ausgewählte Methoden zur Bestimmung von Inhaltsstoffen im Meer-und Brackwasser. Berlin: Nationalkomitee für Geodäsie u. Geophysik bei d. Akad. d. Wiss. d. DDR.

[B60] Rooney-Varga J. N. Giewat M. W. Savin M. C. Sood S. LeGresley M. Martin J. L. (2005). Links between phytoplankton and bacterial community dynamics in a coastal marine environment. Microb. Ecol. 49, 163–175. 10.1007/s00248-003-1057-0 15688258

[B61] Rösel S. Grossart H.-P. (2012). Contrasting dynamics in activity and community composition of free-living and particle-associated bacteria in spring. Aquat. Microb. Ecol. 66, 169–181. 10.3354/ame01568

[B62] Schloss P. D. Westcott S. L. Ryabin T. Hall J. R. Hartmann M. Hollister E. B.. (2009). Introducing mothur: open-source, platform-independent, community-supported software for describing and comparing microbial communities. Appl. Environ. Microbiol. 75, 7537–7541. 10.1128/AEM.01541-09 19801464 PMC2786419

[B63] Simon H. M. Smith M. W. Herfort L. (2014). Metagenomic insights into particles and their associated microbiota in a coastal margin ecosystem. Front. Microbiol. 5:466. 10.3389/fmicb.2014.00466 25250019 PMC4155809

[B64] Simon M. Azam F. (1989). Protein content and protein synthesis rates of planktonic marine bacteria. Mar. Ecol. Prog. Ser. Oldend. 51, 201–213. 10.3354/meps051201

[B65] Simon M. Grossart H. P. Schweitzer B. Ploug H. (2002). Microbial ecology of organic aggregates in aquatic ecosystems. Aquat. Microb. Ecol. 28, 175–211. 10.3354/ame028175

[B66] Smith M. W. Allen L. Z. Allen A. E. Herfort L. Simon H. M. (2013). Contrasting genomic properties of free-living and particle-attached microbial assemblages within a coastal ecosystem. Front. Microbiol. 4:120. 10.3389/fmicb.2013.00120 23750156 PMC3668451

[B67] Sogin M. L. Morrison H. G. Huber J. A. Welch D. M. Huse S. M. Neal P. R.. (2006). Microbial diversity in the deep sea and the underexplored “rare biosphere.” Proc. Natl. Acad. Sci. U.S.A. 103, 12115–12120. 10.1073/pnas.0605127103 16880384 PMC1524930

[B68] Teeling H. Fuchs B. M. Becher D. Klockow C. Gardebrecht A. Bennke C. M.. (2012). Substrate-controlled succession of marine bacterioplankton populations induced by a phytoplankton bloom. Science 336, 608–611. 10.1126/science.1218344 22556258

[B69] Telesh I. V. Khlebovich V. V (2010). Principal processes within the estuarine salinity gradient: a review. Mar. Pollut. Bull. 61, 149–155. 10.1016/j.marpolbul.2010.02.008 20304437

[B70] Urbach E. Vergin K. L. Young L. Morse A. Larson G. L. Giovannoni S. J. (2001). Unusual bacterioplankton community structure in ultra−oligotrophic Crater Lake. Limnol. Oceanogr. 46, 557–572. 10.4319/lo.2001.46.3.0557

[B71] Walsh D. A. Lafontaine J. Grossart H.-P. (2013). On the eco-evolutionary relationships of fresh and salt water bacteria and the role of gene transfer in their adaptation, in Lateral Gene Transfer in Evolution SE - 3, ed Gophna U. (New York, NY: Springer), 55–77.

[B72] Yilmaz P. Parfrey L. W. Yarza P. Gerken J. Pruesse E. Quast C. (2013). The SILVA and “All-species Living Tree Project (LTP)” taxonomic frameworks. Nucleic Acids Res. 41:gkt1209 10.1093/nar/gkt1209 PMC3965112 24293649

[B73] Zeder M. Ellrott A. Amann R. (2011). Automated sample area definition for high−throughput microscopy. Cytom. Part A 79, 306–310. 10.1002/cyto.a.21034 21412981

