# Supplementary figures and images for: Particle-Associated Differ from Free-Living Bacteria in Surface Waters of the Baltic Sea
Source: Front Microbiol. 2015 Dec 1;6:1297. doi: 10.3389/fmicb.2015.01297 (PMC4664634; doi:10.3389/fmicb.2015.01297)

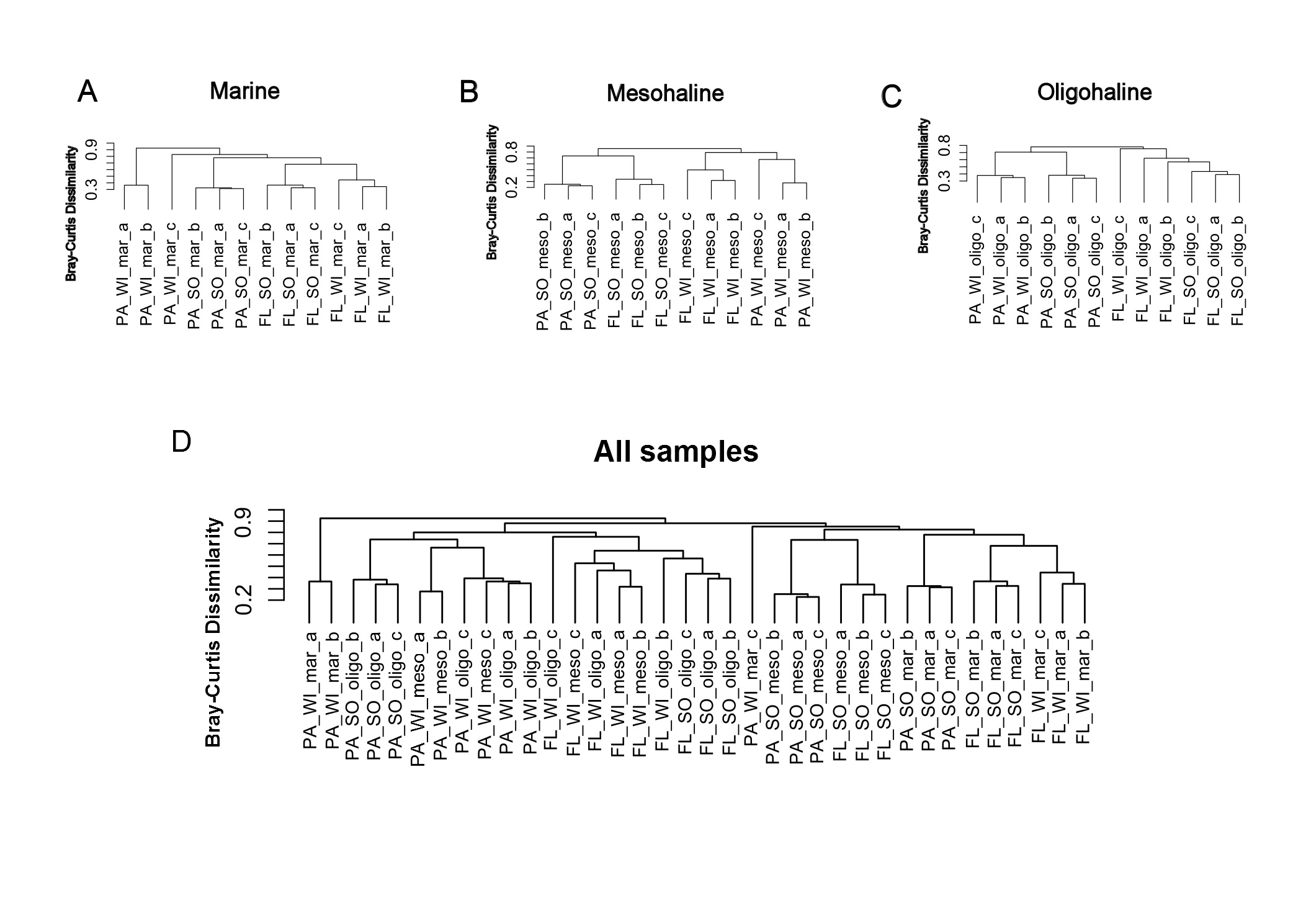

Supplement: Supplemental Figure S1 — Unweighted-pair group method with arithmetic mean (UPGMA) dendograms based on Bray-Curtis dissimilarities of PA and FL bacteria distribution at different stations [marine (A), mesohaline (B), oligohaline (C), and for all samples (D)]. [file FigureS1.JPEG]

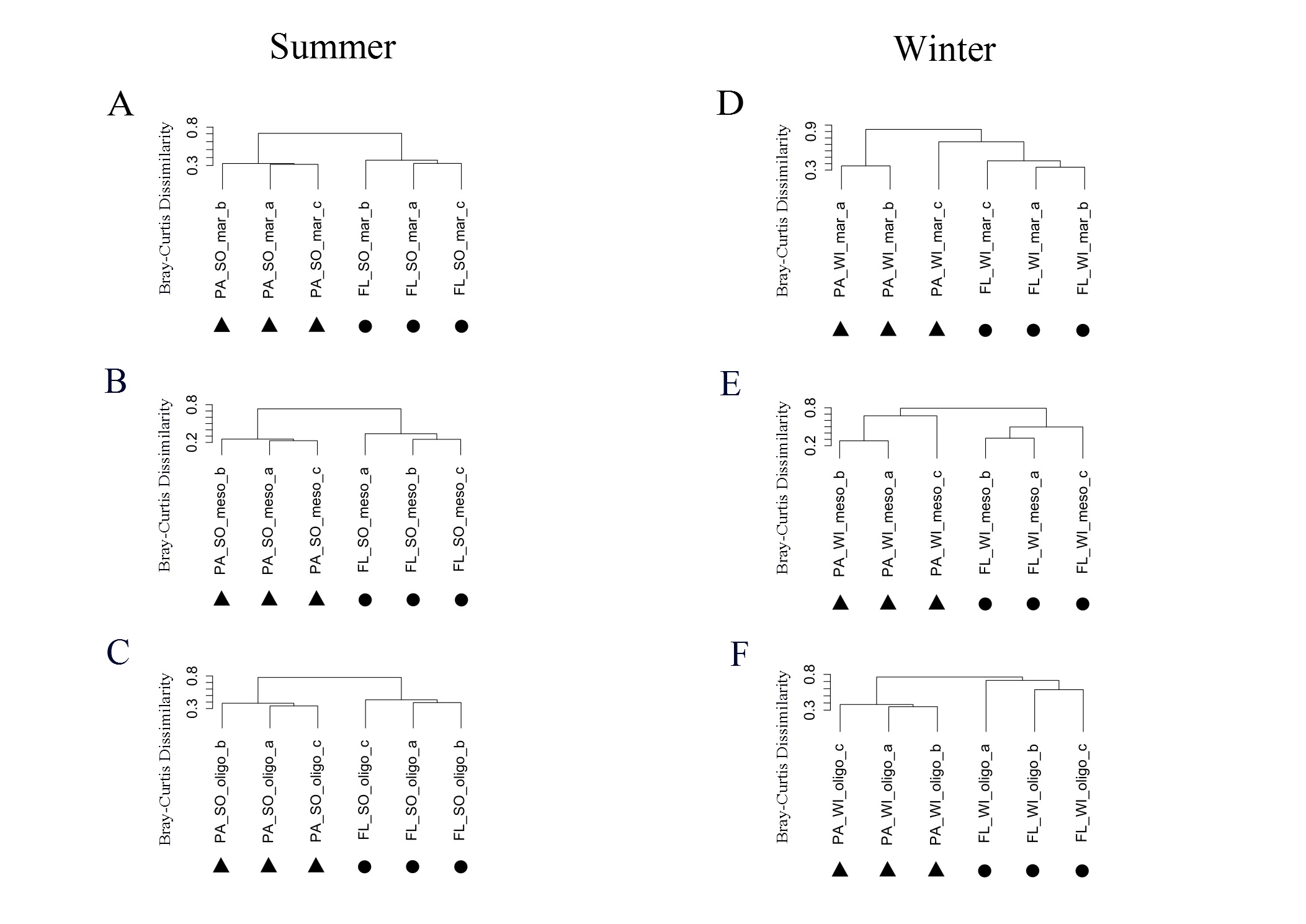

Supplement: Supplemental Figure S2 — Unweighted-pair group method with arithmetic mean (UPGMA) dendograms based on Bray-Curtis dissimilarities of the distribution of PA and FL bacteria at different stations in summer and winter marine (A,D), mesohaline (B,E), oligohaline (C,F). [file FigureS2.JPEG]

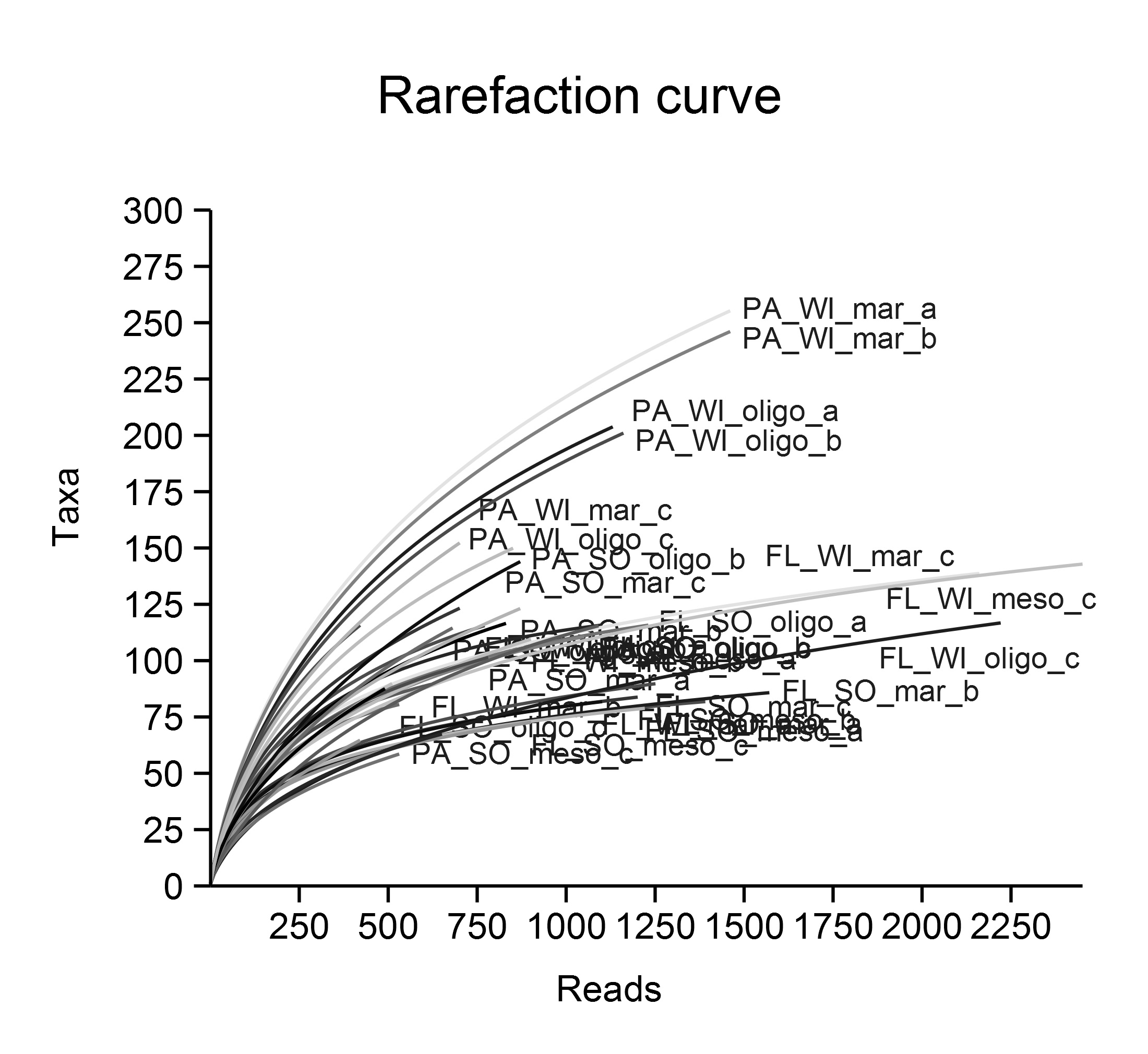

Supplement: Supplemental Figure S3 — Rarefaction curves for free-living and particle-associated samples of the total dataset. [file FigureS3.JPEG]
