# Supplementary material for: Particle-Associated Differ from Free-Living Bacteria in Surface Waters of the Baltic Sea
Source: Front Microbiol. 2015 Dec 1;6:1297. doi: 10.3389/fmicb.2015.01297 (PMC4664634; doi:10.3389/fmicb.2015.01297)
Supplement: Supplemental Table S1 — Wilcoxon Rank sum test between all samples within station and season and a factor (F) from 0 to 1, which compares the groups [triplicates (a, b, c) vs. fractions (PA, FL)] within one station (mar, meso, oligo) and season (summer, fall/winter) to estimate the difference between the fractions, with 1 as highest possible difference. [file TableS1.DOCX]

**Supplemental table S1: Wilcoxon Rank sum test between all samples within station and season and a factor (F) from 0 to 1, which compares the groups (triplicates (a, b, c) vs. fractions (PA, FL)) within one station (mar, meso, oligo) and season (summer, fall/winter) to estimate the difference between the fractions, with 1 as highest possible difference.**

| **Summer** | | | | | | | | | | | **Fall/Winter** | | | | | |
| --- | --- | --- | --- | --- | --- | --- | --- | --- | --- | --- | --- | --- | --- | --- | --- | --- |
| **mar** | | **PA** | | | | **FL** | | | | | **PA** | | | **FL** | | |
|  |  | **A** | | **B** | **C** | **A** | | **B** | | **C** | **A** | **B** | **C** | **A** | **B** | **C** |
| **PA** | **A** |  | | **0.941** | **0.216** | **0.014** | | **0.014** | | 0.122 |  | **0.559** | 0.002 | **0.000** | **0.000** | **0.000** |
|  | **B** |  | |  | **0.216** | **0.008** | | **0.007** | | 0.086 |  |  | 0.010 | **0.000** | **0.000** | **0.000** |
|  | **C** |  | |  |  | **0.000** | | **0.000** | | **0.004** |  |  |  | **0.000** | 0.057 | 0.075 |
| **FL** | **A** |  | |  |  |  | | **0.950** | | **0.355** |  |  |  |  | **0.131** | **0.089** |
|  | **B** |  | | F = **0.867** |  |  | |  | | **0.332** |  | F = **0.733** |  |  |  | **0.860** |
|  | **C** |  | |  |  |  | |  | |  |  |  |  |  |  |  |
|  | | | | | | | | | | | | | | | | |
| **meso** | | **PA** | | | | **FL** | | | | | **PA** | | | **FL** | | |
|  |  | **A** | | **B** | **C** | **A** | | **B** | | **C** | **A** | **B** | **C** | **A** | **B** | **C** |
| **PA** | **A** |  | | **0.243** | **0.376** | 0.874 | | 0.411 | | 0.964 |  | **0.982** | 0.001 | 0.697 | 0.867 | 0.819 |
|  | **B** |  | |  | **0.793** | 0.196 | | 0.054 | | 0.241 |  |  | 0.001 | 0.712 | 0.877 | 0.807 |
|  | **C** |  | |  |  | 0.313 | | 0.104 | | 0.375 |  |  |  | **0.004** | **0.003** | **0.001** |
| **FL** | **A** |  | |  |  |  | | **0.573** | | **0.917** |  |  |  |  | **0.821** | **0.535** |
|  | **B** |  | | F = 0.400 |  |  | |  | | **0.475** |  | F = 0.467 |  |  |  | **0.705** |
|  | **C** |  | |  |  |  | |  | |  |  |  |  |  |  |  |
|  | | | | | | | | | | | | | | | | |
| **oligo** | | | **PA** | | | | **FL** | | | | **PA** | | | **FL** | | |
|  |  |  | **A** | **B** | **C** | | **A** | **B** | **C** | | **A** | **B** | **C** | **A** | **B** | **C** |
| **PA** | **A** | |  | **0.563** | 0.028 | | 0.966 | 0.687 | 0.403 | |  | **0.570** | **0.511** | **0.010** | **0.017** | **0.000** |
|  | **B** | |  |  | 0.005 | | 0.586 | 0.856 | 0.160 | |  |  | **0.220** | **0.002** | **0.003** | **0.000** |
|  | **C** | |  |  |  | | **0.035** | **0.016** | 0.250 | |  |  |  | **0.045** | 0.073 | **0.000** |
| **FL** | **A** | |  |  |  | |  | **0.741** | **0.405** | |  |  |  |  | **0.852** | 0.001 |
|  | **B** | |  | F = 0.400 |  | |  |  | **0.257** | |  | F = **0.800** |  |  |  | 0.000 |
|  | **C** | |  |  |  | |  |  |  | |  |  |  |  |  |  |

**Supplemental table S2: Number of OTUs, reads, Chao1, Shannon [H] and Simpson [1-D] and Buzas and Gibson's Evenness [e^H/S] diversity indexes of averages of samples after normalization to 430 reads per sample. PA: particle-attached. FL: free-living. PA: particle-associated fraction, FL: free-living fraction, Tot: total bacteria.**

|  | | **NormalizedReads**  **N** | **Taxa**  **n** | **Chao1**  **Richness** | **Shannon**  **[H]** | **Simpson**  **[1-D]** | **Evenness**  **[e^H/S]** |
| --- | --- | --- | --- | --- | --- | --- | --- |
| **Summer** | | | | | | | |
| **marine** | **PA** | 430 | 89 | 144.7 | 3.68 | 0.95 | 0.45 |
|  | **FL** | 430 | 65 | 108.9 | 3.37 | 0.94 | 0.45 |
|  | | | | | | | |
| **mesohaline** | **PA** | 430 | 56 | 92.11 | 2.6 | 0.8 | 0.24 |
|  | **FL** | 430 | 67 | 97 | 3.52 | 0.95 | 0.5 |
|  | | | | | | | |
| **oligohaline** | **PA** | 430 | 98 | 195.2 | 3.15 | 0.87 | 0.24 |
|  | **FL** | 430 | 99 | 144 | 3.66 | 0.94 | 0.39 |
| **Fall/Winter** | | | | | | | |
| **marine** | **PA** | 430 | 166 | 324.4 | 4.61 | 0.98 | 0.61 |
|  | **FL** | 430 | 91 | 154.1 | 3.84 | 0.97 | 0.51 |
|  | | | | | | | |
| **mesohaline** | **PA** | 430 | 100 | 196.3 | 3.57 | 0.93 | 0.35 |
|  | **FL** | 430 | 86 | 134.5 | 3.53 | 0.93 | 0.4 |
|  | | | | | | | |
| **oligohaline** | **PA** | 430 | 126 | 203 | 4.05 | 0.96 | 0.46 |
|  | **FL** | 430 | 83 | 112 | 3.01 | 0.85 | 0.24 |
